# Metabolomics analysis identifies sex-associated metabotypes of oxidative stress and the autotaxin–lysoPA axis in COPD

**DOI:** 10.1183/13993003.02322-2016

**Published:** 2017-06-22

**Authors:** Shama Naz, Johan Kolmert, Mingxing Yang, Stacey N. Reinke, Muhammad Anas Kamleh, Stuart Snowden, Tina Heyder, Bettina Levänen, David J. Erle, C. Magnus Sköld, Åsa M. Wheelock, Craig E. Wheelock

**Affiliations:** 1 Division of Physiological Chemistry 2, Dept of Medical Biochemistry and Biophysics, Karolinska Institutet, Stockholm, Sweden; 2 Division of Experimental Asthma and Allergy Research, Institute of Environmental Medicine, Karolinska Instituet, Stockholm, Sweden; 3 Respiratory Medicine Unit, Dept of Medicine Solna and Center for Molecular Medicine, Karolinska Institutet, Stockholm, Sweden; 4 Division of Pulmonary and Critical Care Medicine, Dept of Medicine and Lung Biology Center, University of California San Francisco, San Francisco, CA, USA; 5 Both authors contributed equally

## Abstract

Chronic obstructive pulmonary disease (COPD) is a heterogeneous disease and a leading cause of mortality and morbidity worldwide. The aim of this study was to investigate the sex dependency of circulating metabolic profiles in COPD.

Serum from healthy never-smokers (healthy), smokers with normal lung function (smokers), and smokers with COPD (COPD; Global Initiative for Chronic Obstructive Lung Disease stages I–II/A–B) from the Karolinska COSMIC cohort (n=116) was analysed using our nontargeted liquid chromatography–high resolution mass spectrometry metabolomics platform.

Pathway analyses revealed that several altered metabolites are involved in oxidative stress. Supervised multivariate modelling showed significant classification of smokers from COPD (p=2.8×10^−7^). Sex stratification indicated that the separation was driven by females (p=2.4×10^−7^) relative to males (p=4.0×10^−4^). Significantly altered metabolites were confirmed quantitatively using targeted metabolomics. Multivariate modelling of targeted metabolomics data confirmed enhanced metabolic dysregulation in females with COPD (p=3.0×10^−3^) relative to males (p=0.10). The autotaxin products lysoPA (16:0) and lysoPA (18:2) correlated with lung function (forced expiratory volume in 1 s) in males with COPD (r=0.86; p<0.0001), but not females (r=0.44; p=0.15), potentially related to observed dysregulation of the miR-29 family in the lung.

These findings highlight the role of oxidative stress in COPD, and suggest that sex-enhanced dysregulation in oxidative stress, and potentially the autotaxin–lysoPA axis, are associated with disease mechanisms and/or prevalence.

## Abstract 2

**Oxidative stress and the autotaxin–lysoPA axis evidence sex-associated metabotypes in the serum of COPD patients** http://ow.ly/kAeE309MpdI

## Introduction

Chronic obstructive pulmonary disease (COPD) is an umbrella diagnosis that is characterised by airflow obstruction and permanent reduction of the forced expiratory volume [1]. COPD-related mortality is estimated to reach 1 billion by the end of the 21st century [2]. The early diagnosis of COPD is challenging due to disease heterogeneity and lack of predictive molecular markers. The diagnosis is based solely on spirometry, while lung function, symptoms and exacerbation history are used for disease staging. Decline in lung function over time is accepted as a reliable index of disease progression; however, the mechanisms underlying different COPD subphenotypes and their relationship with prognosis are still unclear [3]. For example, evidence of sex differences, with higher mortality in females even after correction for smoking has emerged [4]. Smoking results in greater impairment in lung function in females, especially post-menopause [5, 6].

Cigarette smoking exerts extensive airway epithelial damage and is an important component driving the onset of COPD [7]. However, not all smokers develop COPD and disease severity varies among smoking COPD individuals. Other risk factors include genetics, asthma, environmental exposures, premature birth and persistent respiratory infections in early childhood [8, 9]. In addition, COPD pathogenesis may be linked to oxidative stress resulting from the overproduction of oxidants/reactive oxygen species (ROS), arising endogenously (*e.g.* from mitochondrial respiration or immune cells) or exogenously (*e.g.* tobacco smoke) [10, 11].

The aim of the current study was to employ a nontargeted high-resolution mass spectrometry (HRMS) metabolomics approach to identify molecular markers of metabolic dysregulation in COPD using the Karolinska COSMIC (Clinical & Systems Medicine Investigations of Smoking-related Chronic Obstructive Pulmonary Disease) cohort [12–15]. A particular focus of the COSMIC study is to evaluate the role of sex in the aetiology of COPD. Accordingly, our statistical analysis focused on sex-specific shifts in the observed metabolic pathways.

## Materials and methods

### Subjects and study design

The Karolinska COSMIC cohort (www.clinicaltrials.gov/ct2/show/NCT02627872) is a three-group cross-sectional study designed for investigating molecular sex differences in early-stage COPD, including 40 never-smokers (“healthy”), 40 smokers with normal lung function (“smokers”) and 38 individuals with COPD (Global Initiative for Chronic Obstructive Lung Disease stage I–II/A–B; forced expiratory volume in 1 s (FEV_1_) 51–97%; FEV_1_/forced vital capacity (FVC) <70%; 27 current smokers (“COPD”) and 11 ex-smokers (“COPD-ExS”)) [12–15]. Of the 118 recruited individuals, two never-smokers did not provide a blood sample and were excluded from the analysis. The remaining 116 subjects were matched for age, sex and current smoking status, and history where relevant (table 1 and online supplementary table E1). Blood was drawn from fasting individuals by venipuncture between 07:00 h and 09:00 h and allowed to stand at room temperature for ≥30 min before centrifugation at 1695*×g* for 10 min at room temperature, and stored at −80°C until use. During the same visit, bronchoalveolar lavage (BAL) was performed and bronchial epithelial cell (BEC) brushings were collected. Detailed methods, as well as study inclusion and exclusion criteria are provided in the online supplementary material. The study was approved by the Stockholm regional ethical board (case number 2006/959-31/1) and participants provided their informed written consent.

**TABLE 1:** Clinical parameters of individuals from the Karolinska COSMIC cohort included in the current study

	**Healthy never-smokers**		**Smokers**		**COPD**		**COPD ex-smokers**	
	**Male**	**Female**	**Male**	**Female**	**Male**	**Female**	**Male**	**Female**
**Subjects**	20	18	20	20	15	12	5	6
**Age years**	62.0 (51.5–64.0)	55.5 (47.8–62.0)	52.5 (49.0–56.0)	54.0 (48.0–58.0)	61.0 (55.0–63.0)	59.0 (57.0–63.0)	64.0 (58.0–65.5)	58.0 (53.8–65.0)
**BMI kg·m^-2^**	25.6 (23.5–27.9)	26.5 (23.3–30.6)	25.0 (21.9–26.2)	24.2 (22.6–25.9)	24.2 (21.3–28.7)	23.5 (20.8–26.0)	29.1 (24.0–31.0)	27.6 (22.3–29.6)
**Smoking pack-years**	NA	NA	33.5 (30.0–40.0)	33.0 (27.3–40.0)	42.0 (36.0–50.0)	40.5 (35.8–47.3)	30.0 (21.5–39.5)	28.5 (19.3–37.8)
**Menopause no/yes**	NA	12/6	NA	8/12	NA	0/12	NA	1/5
**GOLD stage 1/2**	NA	NA	NA	NA	7/7	6/6	2/3	4/2
**GOLD-2011 A/B/C**	NA	NA	NA	NA	11/4/0	9/3/0	3/1/1	4/2/0
**Blood leukocytes ×10^9^·L^-1^**	5.8 (4.8–6.7)	5.6 (5.0–6.8)	7.4 (6.9–8.3)	6.8 (6.3–8.0)	7.8 (6.4–9.2)	8.2 (5.9–10.2)	6.6 (5.5–7.5)	7.0 (6.5–9.3)
**Blood platelets ×10^9^·L^-1^**	216.0 (193.3–246.8)	267.5 (244.5–307.8)	239.0 (209.0–272.0)	287.5 (241.5–346.3)	264.0 (224.0–345.0)	280.5 (235.8–329.5)	199.0 (196.0–285.0)	244.5 (207.0–327.8)
**Serum albumin g·L^−1^**	40.0 (38.0–42.0)	40.0 (37.8–41.0)	39.0 (38.0–41.0)	39.0 (37.3–39.8)	38.0 (37.0–38.0)	39.5 (38.0–41.0)	38.0 (36.5–39.5)	41.0 (39.3–42.0)
**Antitrypsin g·L^−1^**	1.4 (1.3–1.5)	1.4 (1.3–1.5)	1.4 (1.3–1.6)	1.6 (1.4–1.7)	1.6 (1.4–1.7)	1.6 (1.4–1.7)	1.4 (1.3–1.7)	1.4 (1.2–1.6)
**FEV**_**1**_ **%**	119.0 (104.0–127.5)	120.5 (111.0–127.3)	107.0 (103.3–118.5)	110.0 (98.3–116.0)	78.0 (73.0–84.0)	78.5 (74.3–93.5)	72.0 (58.0–91.0)	83.5 (73.8–90.8)
**FEV**_**1**_**/FVC %**	80.0 (76.3–84.8)	82.5 (76.8–84.3)	77.0 (74.3–80.0)	79.0 (74.3–82.5)	64.0 (56.0–66.0)	61.5 (53.8–63.5)	59.0 (48.5–68.0)	64.0 (56.5–66.3)
**Emphysema no/yes**	NA	NA	10/10	7/13	5/10	1/11	1/4	4/2
**Chronic bronchitis no/yes**	NA	NA	13/7	13/7	13/2	7/5	1/4	5/1

Data are presented as n or median (interquartile range). COPD: chronic obstructive pulmonary disease; BMI: body mass index; GOLD: Global Initiative for Chronic Obstructive Lung Disease; FEV_1_: forced expiratory volume in 1 s; FVC: forced vital capacity; NA: not applicable.

### Mass spectrometry analysis

Sample processing and analyses were performed as previously published [16] and are described in the online supplementary material. Briefly, for nontargeted metabolomics, 50 μL of serum was used for both hydrophilic interaction liquid chromatography (HILIC) and reversed-phase (RP) chromatography. Samples were analysed using an Ultimate 3000 UHPLC coupled to a Q-Exactive Orbitrap mass spectrometer (Thermo Fisher Scientific, Bremen, Germany). Mass spectrometry (MS) data were acquired (full scan mode) in both positive and negative ionisation. Molecular features were extracted using the software XCMS (https://metlin.scripps.edu). Putative metabolite annotation was performed using the Human Metabolome Database (HMDB) [17], and output matched to an in-house accurate mass/retention time library of reference standards [18]. Metabolite identity was described as confirmed following a match to reference standards and/or MS/MS. Targeted metabolite quantification was performed using the Biocrates AbsoluteIDQ p180 kit (Biocrates Life Sciences, Innsbruck, Austria) on a Xevo TQ-S triple quadrupole (Waters Corporation, Milford, MA, USA).

### miRNA profiling

miRNA from BAL cells and BECs, and exosomes from BAL fluid from a subset of the COSMIC cohort based upon sample availability (n=45; five to 13 subjects per group and sex) were analysed as described in the online supplementary material. Statistical analyses were performed on probe intensities from a subset of four miRNAs of interest, selected using TargetScan release 7.1 (June 2016): miR-29a-3p, miR-29b-3p, miR-29c-3p targeting autotaxin (ENPP2) and miR-218-5p targeting *N*-acyl phosphatidylethanolamine phospholipase D (NAPE-PLD).

### Statistical analysis

Due to the confounding effects of smoking, stratification by smoking status was applied in both univariate and multivariate statistical analyses. Accordingly, the smoking population (smokers and COPD) and nonsmoking population (healthy and COPD-ExS) were analysed separately. Statistical analysis was applied to metabolites present in ≥70% of the samples in at least one group, with a coefficient of variation <30% in quality control samples [19]. The percentage of missing values was compared across all clinical groups prior to removal, to ensure that a metabolite was not erroneously removed due to being absent completely in one or more groups. Metabolites with a quality control relative standard deviation >25% were deemed not suitably reproducible and removed from further analysis; this value was chosen based on literature reports [20] and our choice of chromatography (RP and HILIC). Four samples were not analysed in HILIC positive mode due to lack of material, for which missing values were imputed using k-nearest neighbours (k=10) imputation [21].

Univariate statistical analysis was performed on filtered data using the Mann–Whitney test and the Storey q-value (MATLAB vR2015a; MathWorks, Natick, MA, USA). Correction for p-values with regards to age and smoking history between smokers and COPD groups was performed in STATA (v12; StataCorp, College Station, TX, USA) (online supplementary table E2).

Multivariate statistical modelling was performed on log-transformed, mean-centered and pareto-scaled data using SIMCA (v14.0; MKS Umetrics, Malmö, Sweden). Orthogonal projections to latent structures discriminant analysis (OPLS-DA) was performed using metabolites that passed quality control. OPLS-DA models were optimised using variable selection criteria of |p(corr)|≥0.4 (loadings scaled as correlation coefficient between model and original data) and variable importance in projection ≥1.0 as previously described [22]. Model statistics are reported by the cumulative correlation coefficient (R^2^Y), the predictive variance based on seven-fold cross-validation (Q^2^) and cross-validated ANOVA p-values for OPLS models. Shared-and-unique-structure (SUS) analysis correlating p(corr) values between models was performed as previously described [23]. A short tutorial on the multivariate methods is provided in the online supplementary material. Pathway enrichment analysis on structurally confirmed metabolites was performed using integrated pathway-level analysis [24]. In addition, we performed stratification by sex prior to univariate and multivariate statistical analyses to facilitate investigation of inter- and intra-group sex differences. Additionally, investigations of the effects of menopause were performed by construction of multivariate models including and excluding premenopausal females, and correlating these models through SUS-based analysis.

## Results

### Smokers *versus* COPD using nontargeted metabolomics

#### Univariate statistical data analysis

A metabolite is described as “putative” following an accurate mass match to the HMDB database [17]. A metabolite is described as “confirmed” following a match to reference standards and/or MS/MS spectrum. A total of 1153 putative metabolites were extracted from the nontargeted metabolomics raw data, of which 959 passed quality control. These putative metabolites were subjected to both sex-combined and sex-stratified comparisons of smokers *versus* COPD. Of the 959 putative metabolites, 184 were significant at p<0.05 and selected for structural confirmation. Of these 184 metabolites, 67 were structurally confirmed by MS/MS and/or matching to reference standards, and the corresponding p-value*,* Storey's q-value and fold change are provided in online supplementary table E2. All nontargeted metabolomics data presented in this study refer to these 67 structurally confirmed metabolites.

#### Multivariate statistical modelling

**FIGURE 1:**
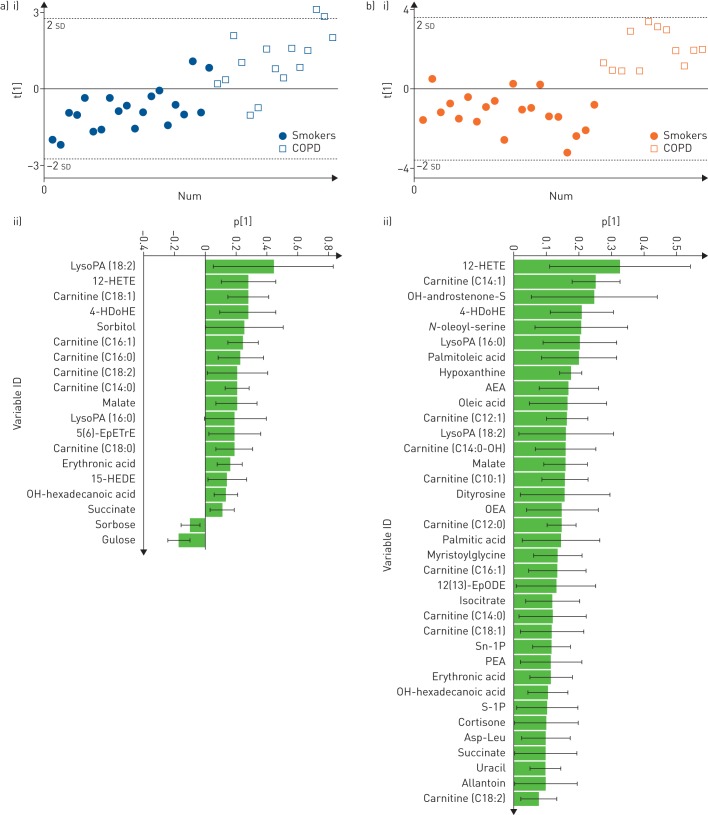
Optimised orthogonal projections to latent structures discriminant analysis multivariate models using nontargeted metabolomics data. a) i) Scores plot of male smokers *versus* males with chronic obstructive pulmonary disease (COPD) (R^2^Y=0.49, Q^2^=0.38, p=4.0×10^−4^); ii) loadings of confirmed metabolites that were the most prominent for driving the separation between male smokers *versus* males with COPD. b) i) Scores plot of female smokers *versus* females with COPD (R^2^Y=0.73, Q^2^=0.65, p=2.4×10^−7^); ii) loadings of confirmed metabolites that were the most prominent for driving the separation of female smokers *versus* females with COPD. For ease of display, parts a ii) and b ii) exclude metabolites whose standard error crossed the x-axis. The complete list of loadings is shown in online supplementary figure E8. LysoPA: lyso-phosphatidic acid; HETE: hydroxyeicosatetraenoic acid; HDoHE: hydroxydocosahexaenoic acid; EpETrE: epoxyeicosatrienoic acid; HEDE: hydroxyeicosadienoic acid; AEA: *N*-arachidonoylethanolamine; OEA: *N*-oleoylethanolamine; EpODE: epoxyoctadecadienoic acid; PEA: *N*-palmitoylethanolamide; Asp-Leu: aspartic acid-leucine.

Multivariate statistical modelling of smokers *versus* COPD was performed using OPLS-DA on the 67 confirmed metabolites. The joint sex OPLS-DA model for classifying smokers *versus* COPD showed significant group separation (R^2^Y=0.45, Q^2^=0.38, p=2.8×0^−7^; online supplementary figure E1), with a receiver operating characteristic area under the curve (AUC) of 0.90. Sex stratification revealed that the group separation in smokers *versus* COPD was more robust in females (R^2^Y=0.73, Q^2^=0.65, p=2.4×0^−7^, AUC=1.0) relative to males (R^2^Y=0.49, Q^2^=0.38, p=4.0×0^−4^, AUC=0.89) (figure 1). Permutation tests confirmed the robustness of the models (Y-intercept (500 permutations): females R^2^Y=0.24, Q^2^=−0.18; males R^2^Y=0.20, Q^2^=−0.26; figure not shown). Only 13 metabolites overlapped all three models (online supplementary figure E2).

**FIGURE 2:**
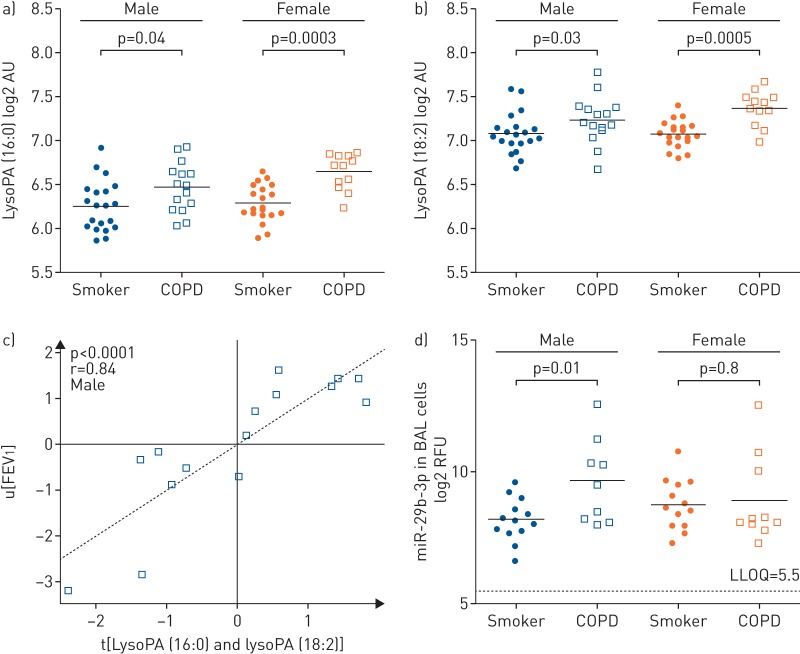
The lyso-phosphatidic acid (lysoPA)–autotaxin axis was attenuated in males with chronic obstructive pulmonary disease (COPD). a) Serum lysoPA (16:0) levels in smokers *versus* COPD; (b) serum lysoPA (18:2) levels in smokers *versus* COPD; (c) lysoPA (16:0) and lysoPA (18:2) metabolites correlated with lung function (forced expiratory volume in 1 s (FEV_1_)) in male COPD patients (r=0.84, p<0.0001). No correlation was observed in the corresponding female COPD population (r=0.44, p=0.15); (d) levels of miR-29b in bronchoalveolar lavage (BAL) cells from male and female smokers and COPD patients. Values for the other members of the miR-29 family are shown in online supplementary figure E6. LysoPA data are from the nontargeted metabolomics platform and are presented as log2 of arbitrary units (AU). RFU: relative fluorescence units; LLOQ: lower limit of quantification.

Correlations were performed for all significantly altered metabolites with lung function parameters (FEV_1_ (%) and FEV_1_/FVC) using Spearman's correlation, as well as group-wise using partial least squares multivariate correlation (online supplementary figure E3). Lysophosphatidic acid (lysoPA) (16:0) and lysoPA (18:2) were most strongly correlated with FEV_1_ (%), and were further stratified by sex, evidencing strong correlations in male COPD patients (partial least squares inner relation: r=0.86, p<0.0001) (figure 2), but not females (r=0.44, p=0.15). Based upon these findings, the serum levels of lysoPA (16:0) and lysoPA (18:2) were examined and found to exhibit greater increases in females with COPD relative to smokers (p=0.0003 and p=0.0005, respectively) than the corresponding males (p=0.04 and p=0.03, respectively) (figure 2).

All females in the COPD group were postmenopausal, while 40% (n=8) female smokers were premenopausal. To investigate the role of menopausal status, OPLS-DA models including only postmenopausal subjects were constructed, and correlated with the original model based on all female subjects using SUS analyses. The high correlation between the two models (R^2^=0.92) indicates that no substantial differences in metabolite levels were observed due to menopausal status (online supplementary figure E4).

#### Pathway enrichment analysis

Pathway analysis of the COPD-associated metabolic perturbations from the nontargeted metabolomics data identified significant shifts (p≤0.05) in eight biochemical pathways (table 2), with COPD-associated increase in metabolites of the tricarboxylic acid (TCA) cycle, glycerophospholipids, cAMP signalling, endocannabinoids, sphingolipid and fatty acid metabolism. Sex-stratified pathway analyses established that the fatty acid and sphingolipid pathways were enhanced in females, whereas shifts in cAMP signalling and endocannabinoid and tryptophan metabolism pathways were enhanced in males. The altered metabolic changes based upon the pathway analysis also highlight a strong state of oxidative stress in COPD.

**TABLE 2:** Metabolic pathways significantly altered in chronic obstructive pulmonary disease (COPD)^#^

**Pathway name(s)**	**Number of metabolites in pathway**	**Smokers *versus* COPD**					
		**Both sexes**		**Female**		**Male**	
		**Hits**	**p-value (FDR**^¶^**)**	**Hits**	**p-value (FDR**^¶^**)**	**Hits**	**p-value (FDR**^¶^**)**
**Alterations in both sexes**							
Citrate (tricarboxylic acid) cycle		3	0.0009 (0.005)	3	0.0006 (0.005)	2	0.005 (0.01)
Glycerophospholipid metabolism	52	3	0.002 (0.009)	3	0.002 (0.007)	4	<0.0001 (0.0001)
Pyruvate metabolism	31	2	0.03 (0.05)	2	0.03 (0.04)	2	0.01 (0.02)
**Sex-enhanced: female COPD**							
Fatty acid biosynthesis	50	3	0.0002 (0.002)	2	0.006 (0.01)	0	1.0 (1.0)^+^
Sphingolipid metabolism	25	2	0.02 (0.05)	2	0.02 (0.03)	0	1.0 (1.0)^+^
**Sex-enhanced: male COPD**							
cAMP signalling pathway	40	2	0.03 (0.05)	2	0.02 (0.03)	2	0.009 (0.02)
Retrograde endocannabinoid signalling	19	2	0.02 (0.04)	2	0.01 (0.02)	2	0.005 (0.02)
Tryptophan metabolism	80	2	0.5 (1.0)^+^	0	1.0 (1.0)^+^	2	0.04 (0.05)

FDR: false discovery rate. ^#^: pathway analysis was performed using integrated pathway-level analysis [24]; ^¶^: calculated using Benjamini–Hochberg method with a cut-off value of p<0.3; ^+^: pathways did not pass the FDR cut-off value.

### Confirmation of oxidative stress results by targeted MS

Metabolites related to oxidative stress were identified as one of the primary drivers for differentiating the smokers and COPD groups (online supplementary table E2). A targeted MS platform (Biocrates AbsoluteIDQ p180 kit) was applied to confirm this finding. Among the 188 metabolites analysed, nine were excluded from further statistical analysis due to ≥70% missing values and/or values below the limit of detection. Measured concentrations of each metabolite as well as the corresponding p-value and q-value are shown in online supplementary table E3. The greatest differences were observed for the female comparisons (smokers *versus* COPD, 26 metabolites p<0.05) relative to the males (smokers *versus* COPD, 11 metabolites p<0.05), confirming the results of the nontargeted metabolomics platform.

OPLS-DA analysis based on the targeted data confirmed significant separation between the smokers and COPD groups (R^2^Y=0.29, Q^2^=0.19, p=2.0×10^−3^). Sex stratification confirmed that the separation between smokers and COPD was driven by the female population (R^2^Y=0.45, Q^2^=0.34, p=3.0×10^−3^), with no significant model for males (p=0.10) (online supplementary figure E5).

**FIGURE 3:**
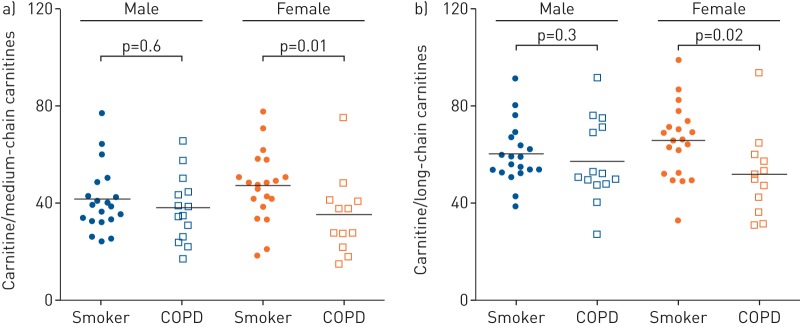
β-oxidation-related metabolite ratio of carnitine with acylcarnitines in relation to sex and disease status for smoking subjects. a) Ratio of carnitine with sum of the medium-chain carnitines; (b) ratio of carnitine with sum of the long-chain carnitines. Subjects are divided into smokers with normal lung function and smokers with chronic obstructive pulmonary disease (COPD). Significance was calculated using the nonparametric Mann–Whitney test. Data are from the targeted metabolomics method (Biocrates).

The relative level of fatty acid β-oxidation was estimated by the ratio of carnitine to acylcarnitine using the sum of short-, medium- and long-chain carnitines. The ratios between medium- and long-chain carnitines were significantly downregulated in the female COPD group *versus* smokers (p=0.01 and p=0.02, respectively), but not in the corresponding male population (figure 3a and b).

**FIGURE 4:**
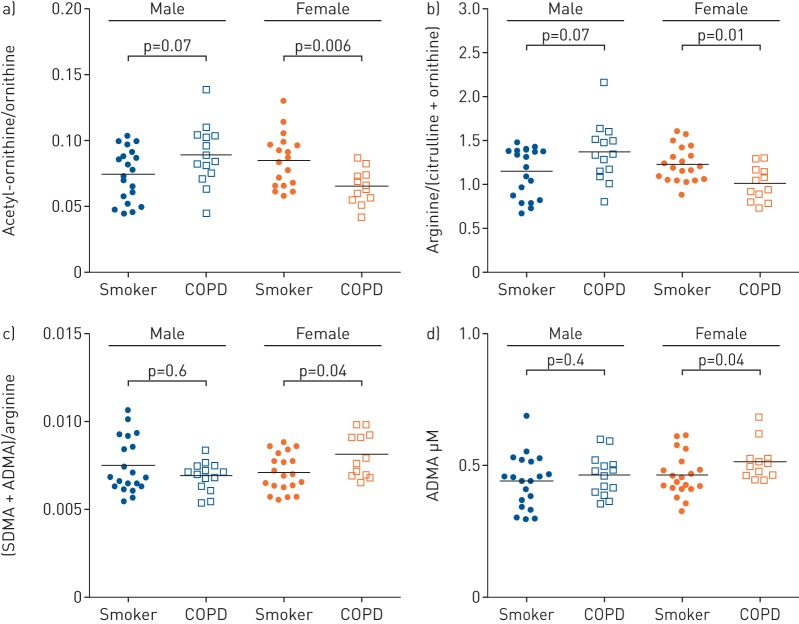
Serum levels of analytes involved in the arginine/nitric oxide pathway. a) Ratio of acetyl-ornithine to ornithine; (b) ratio of total arginine to the inferred activity of the nitric oxide synthase (NOS) enzyme expressed as arginine/(ornithine+citrulline); (c) ratio of endogenous NOS inhibitors (sum of asymmetric and symmetric dimethylarginine (ADMA + SDMA)) with arginine; and (d) concentration of the endogenous NOS inhibitor ADMA. Significance was calculated using the nonparametric Mann–Whitney test. Subjects are divided into smokers with normal lung function and smokers with chronic obstructive pulmonary disease (COPD). Data are from the targeted metabolomics method (Biocrates).

Perturbations in nitric oxide synthesis were examined *via* metabolites of the arginine pathway. The ratios of acetyl–ornithine/ornithine and arginine/(citrulline+ornithine) were significantly lower in females with COPD *versus* smokers (p=0.006 and p=0.01, respectively; figure 4a and b), but not the corresponding male subjects. Conversely, the ratio of asymmetric (ADMA) and symmetric dimethylarginine (SDMA) to arginine as well as ADMA alone was significantly upregulated in females with COPD (p=0.04 and p=0.04, respectively; figure 4c and d), with no differences in male subjects.

### Correlation to miRNA expression in the lung

Aberrant expression of miRNAs has been associated with several pulmonary disorders, including COPD [25–27]. We therefore performed microarray profiling of the miR-29-3p family (-29a, -29b and -29c), which are putative regulators of autotaxin (lysophospholipase D, ENPP2). We found that these miRNAs were present at levels substantially above the limit of detection in BAL cells and BECs in both the smokers and COPD groups (average expression level 2^8^–2^11^), but not in exosomes isolated from BAL fluid. The miR-29 family was significantly upregulated in male COPD patients compared to smokers both in BAL cells (p=0.004–0.056, fold change 1.5–2.7; figure 2d and online supplementary figure E6) and BECs (p=0.03–0.06, fold-change 2.0–2.8, online supplementary figure E6), while no alteration was detected in the corresponding female cohort (BAL p=0.78–0.90; BEC p=0.22–0.29). Levels of miR-218-5p, a putative regulator of NAPE-PLD (an alternative route of lysoPA production) previously reported to be involved in the pathogenesis of COPD [27], were below the lower limit of quantification in all three lung compartments (BAL cells, BECs and BAL fluid exosomes).

### Healthy *versus* COPD-ExS

An OPLS-DA model comparing the nonsmoking population (healthy *versus* COPD-ExS) was correlated to the OPLS-DA model of smokers *versus* COPD groups described earlier, to investigate whether the metabolite shifts related to COPD were independent from current smoking status. SUS correlation analysis between the models describing the nonsmoking and smoking populations was highly correlated (R^2^=0.73), suggesting that the alterations observed due to COPD in the smoking population are independent of current smoking status (online supplementary figure E7).

## Discussion

The objective of the current study was to investigate systemic shifts in metabolism in early-stage COPD. Using our suite of HRMS-based nontargeted and targeted metabolomics platforms, we observed systemic molecular shifts in serum from smokers and early-stage COPD patients. Further stratification revealed sex-associated metabotypes, with a subset of metabolites significantly separating female smokers and female COPD patients (p=2×10^−7^). This corresponds well with our previous findings of a female-associated molecular subphenotype of COPD in this cohort [12, 15].

**FIGURE 5:**
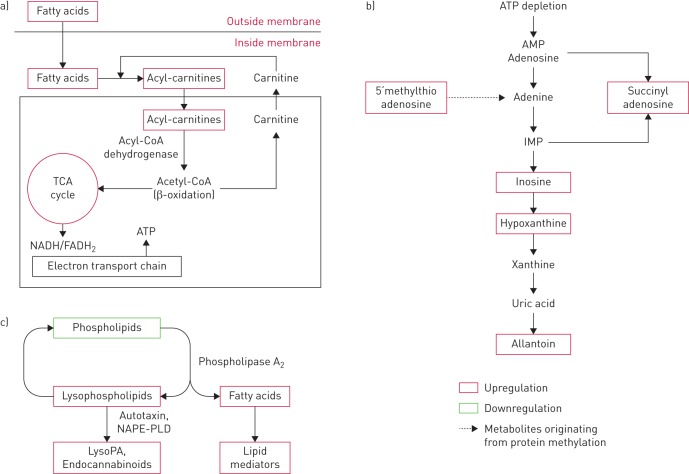
Representative pathway outline for the altered metabolites involved in oxidative stress metabolism in chronic obstructive pulmonary disease (COPD). (a) Fatty acid β-oxidation pathway; (b) purine degradation pathway; (c) Land's cycle/phospholipid metabolism. TCA: tricarboxylic acid; NADH: nicotinamide adenine dinucleotide; FADH_2_: flavin adenine dinucleotide; IMP: inosine monophosphate; NAPE-PLD: *N*-acyl phosphatidylethanolamine phospholipase D; lysoPA: lysophosphatidic acid.

The majority of the observed COPD-related metabolic shifts were associated with oxidative stress (figure 5). The lungs are constantly exposed to ROS, and dysregulation of oxidative stress related pathways has been implicated in airway disease [28, 29]. The observed elevation in circulating levels of acylcarnitines in COPD suggests an amplified energy demand that is reflected in the increased transfer of acetyl coenzyme A to the TCA cycle (figure 5a). In this capacity, free carnitine acts as a fatty acid carrier between the mitochondria and cytosol, and reduced levels of free carnitine in lung tissue have been reported to associate with progressive emphysema [30]. Upregulation of the TCA cycle leads to increased ATP production (figure 5a), and increased extracellular ATP levels in the airway lumen have been associated with COPD pathogenesis *via* the recruitment and activation of inflammatory cells, accelerating inflammation and tissue degradation [31].

Following sex stratification, we observed that the majority of the oxidative stress related shifts were more pronounced in females with COPD. These findings were confirmed by targeted analysis, identifying sex-associated metabotypes of COPD. It has been postulated that antioxidant genes are downregulated in smoking-induced COPD in females. In an elegant mouse study, Tam *et al.* [32] showed that long-term exposure to smoking was associated with increased small airway remodelling and distal airway resistance, as well as downregulation of a range of antioxidant genes and increased oxidative stress in female, but not male or ovariectomised mice. These effects were attenuated by tamoxifen treatment, indicating that female sex hormones play an important role in the sensitivity to smoking, with impairment of antioxidant defence being a contributing factor. In our study, enhanced β-oxidation, purine degradation and endocannabinoid production, as well as the ratios of free carnitine to medium- and long-chain acylcarnitines were significantly increased in females relative to males (figure 3a and b). These findings provide a strong molecular signature that substantiates the findings of Tam *et al.* [32], further supporting the theory of systemic dysregulation of the antioxidant defence in a female-dominated COPD subphenotype [12, 15]. Reactive nitrogen species (RNS) also contribute to oxidative damage in COPD. The arginine pathway is one of the major sources of RNS and is involved in maintaining airway tone [33]. ADMA and SDMA are endogenous nitric oxide synthase (NOS) inhibitors that are associated with COPD prognosis and airway remodelling [34, 35] as well as airway obstruction in asthma [36]. The observed sex-selective alterations in the arginine pathway metabolites (figure 4) suggest that in female COPD, oxidative damage is both ROS- and RNS-mediated. These findings further support the hypothesis that nitrosative stress may be involved in the progression of COPD, with endothelial NOS expression previously reported to increase in the bronchial submucosa of smokers [37].

While a number of metabolites shifted between smokers and COPD, both lysoPA species correlated strongest with lung function (online supplementary figure E3). The enzyme autotaxin (lysophospholipase D) is the primary source of lysoPA lipid mediators in blood [38], and has been suggested as a promising target for COPD treatment [39]. The serum lysoPA levels only correlated with lung function in male COPD patients (figure 2c), suggesting a sex-associated dysregulation in the autotaxin–lysoPA pathway. These findings were supported by greater increases in the levels of autotaxin-regulating miRNA in BAL cells and BECs of male COPD patients relative to females (figure 2d). Interestingly, levels of miR-29b-3p, the family member with the highest alterations, correlated with FEV_1_ in male COPD patients (r=0.62, p=0.07; data not shown), but not female COPD patients (r=0.48, p=0.16), male smokers (r=0.33, p=0.33) or female smokers (r=0.25, p=0.75). The miR-29 family was selected for investigation based upon a TargetScan query for autotaxin; however, there are no reports of miR-29 interacting with autotaxin in the literature, suggesting that this is a new area of investigation. While we observed a strong upregulation in BAL cells and BECs in male COPD patients for the entire miR-29 family, a decrease in miR-29-b has been previously reported in BAL cells from COPD patients [25]. However, the previous study did not register or control for glucocorticoid treatment and the authors reported that it is likely that the COPD patients in their cohort were taking inhaled corticoids (ICS). In the Karolinska COSMIC cohort ICS use was not permitted, with a minimum 3-month washout period. Solberg *et al.* [40] reported 1.7–2.8-fold decreases of all three miR-29s in ICS-using asthmatics compared to healthy controls. Accordingly, the observed discrepancies in miR-29 levels are probably due to differences in ICS treatment. While the role of miR-29 in COPD is unclear, it has been shown to have important functions in pulmonary fibrosis [41] and lung cancer [42, 43], suggesting that it plays a role in lung injury and highlighting the interest in targeting this pathway.

Upregulation of the autotaxin–lysoPA axis has been associated with a number of inflammatory lung conditions, including hyperoxic lung injury [44], fibrosis [45] and asthma [46, 47]. Sex differences in the autotaxin–lysoPA axis have been reported, with both autotaxin [48] and lysoPA [49] plasma levels higher in females relative to males. Platelet activation can also lead to increased serum lysoPA levels *via* autotaxin activity [38], and platelet involvement is supported by the observed increased in serum levels of the 12-lipoxygenase product 12-hydroxyeicosatetraenoic acid (12-HETE; q=0.01 in females, q=0.3 in males). 12-HETE plays a critical role in platelet aggregation and thrombosis [50, 51]. Based on these observations, it is possible that the upregulation of miR-29 exhibits a protective role against oxidative stress-mediated shifts in the autotaxin–lysoPA axis in male, but not female COPD patients, potentially in combination with increased platelet activation in females. However, the potential interaction between miR-29 levels in the lung and circulatory lysoPA levels is unclear. Exosomal regulation is one potential mechanism; however, miR-29 exosome levels were below the limit of quantification. In order to investigate the potential mechanism, further work should determine autotaxin levels in the circulation and the airways as well as quantify the full panel of lysoPA species in both compartments. It would be of particular interest to examine the autotaxin mRNA levels as well as protein levels in BAL cells in order to better understand the relationship between message, protein and metabolite profiles. The current findings suggest that the autotaxin–lysoPA should be further investigated in the pathobiology of COPD, but studies should be designed for sex-stratification.

This study highlights a number of interesting metabolic shifts with COPD and sex; however, there are limitations that should be considered when interpreting the results. While the Karolinska COSMIC cohort is a large study with regards to the invasive sampling through bronchoscopy, from a statistical standpoint the group sizes are relatively small. Accordingly, even though the cross-validated multivariate models gave robust classification models, an independent validation cohort is required to confirm our findings. Furthermore, given our choice to only include confirmed metabolites in the analysis, it is likely that other metabolic shifts occur in the pathobiology of COPD that are not observed with the current metabolite panel. In addition, the longitudinal stability of these metabolite signatures needs to be confirmed. Importantly, the relationship between miRNA levels in the lung compartment and lysoPA levels in circulation is unclear, and as lysoPA can also be released from platelets during the clotting process, this mechanistic information should be interpreted with caution.

To summarise, this study highlights the role of oxidative stress in the pathobiology of COPD. Of particular interest is that even in early-stage COPD, strong systemic alterations were observed in oxidative stress associated metabolic pathways. These findings further highlight the sex differences in COPD, emphasising the importance of sex-stratification in future studies. While oxidative stress appears to be more strongly upregulated in females with COPD, as previously reported [32] the effects may be due to an increase in the antioxidant pathways in the corresponding male population. For example, the selective increase in miR-29b in two lung compartments in males could potentially account for the observed sex differences in the autotaxin–lysoPA axis and its associated pathology. In addition, it has recently been reported that autotaxin binds to steroids [52], further opening the potential for interactions between sex hormones and the autotaxin–lysoPA axis. Finally, as with the previous studies from the Karolinska COSMIC cohort, the majority of the observed alterations were more pronounced in the female population, providing further molecular evidence of a female-driven subphenotype of COPD.

## Supplementary material

10.1183/13993003.02322-2016.Supp1 **Please note:** supplementary material is not edited by the Editorial Office, and is uploaded as it has been supplied by the author.
Supplementary material ERJ-02322-2016_Supplement

## Disclosures

10.1183/13993003.02322-2016.Supp2 A. Wheelock ERJ-02322-2016_A_Wheelock
