# Supplementary material for: Metabolomics analysis identifies sex-associated metabotypes of oxidative stress and the autotaxin–lysoPA axis in COPD
Source: Eur Respir J. 2017 Jun 22;49(6):1602322. doi: 10.1183/13993003.02322-2016 (PMC5898938; doi:10.1183/13993003.02322-2016)
Supplement: Supplementary file 1 [file ERJ-02322-2016_Supplement.pdf]

Supplementary Material

Metabolomics analysis identifies gender-associated metabotypes of oxidative stress and the autotaxin-lysoPA axis in COPD

Shama Naz, PhD[1], Johan Kolmert, MSc[1], Mingxing Yang, MD, PhD[2], Stacey N. Reinke, PhD[1], Muhammad Anas Kamleh, PhD[1], Stuart Snowden, PhD[1], Tina Heyder, MSc[2], Bettina Levänen, PhD[2], David J. Erle, MD[3], C. Magnus Sköld, MD, PhD[2], Åsa M. Wheelock, PhD[2,4,*], Craig E. Wheelock, PhD[1,4,*]

[1]Division of Physiological Chemistry 2, Department of Medical Biochemistry and Biophysics, Karolinska Institutet, Stockholm, Sweden
[2]Respiratory Medicine Unit, Department of Medicine Solna & Center for Molecular Medicine, Karolinska Institutet, Stockholm, Sweden
[3]Division of Pulmonary and Critical Care Medicine, Department of Medicine and Lung Biology Center, University of California San Francisco, San Francisco, USA
[4]Both authors contributed equally

*Correspondence to be addressed to:
Craig E. Wheelock, PhD
Division of Physiological Chemistry 2
Department of Medical Biochemistry and Biophysics
Karolinska Institutet, 17177 Stockholm, Sweden
Email: craig.wheelock@ki.se
Phone: +46 8 524 87630, fax: +46 8 736 0439

or

Åsa Wheelock, PhD
Lung Research Lab L4:01, Respiratory Medicine Unit & Center for Molecular Medicine, Department of Medicine,
Karolinska Institutet, 17176 Stockholm, Sweden
Email: asa.wheelock@ki.se
Phone: +46 8 517 70664, fax: +46 8 517 75451

Materials and Methods:

Subjects and study design

This study examined subjects from the Karolinska COSMIC cohort (www.clinicaltrials.gov/ct2/show/NCT02627872). The COSMIC study is a three group cross sectional study in which each group was stratified by gender with the aim of investigating the differentiation between the genders in early stage COPD [1-4]. A total of 40 never-smokers, 40 smokers with normal lung function and 38 patients with COPD were recruited with the intent to collect peripheral blood. Of the 118 recruited individuals, two never smokers did not provide a blood sample and were excluded from the analysis. The study was accordingly performed on 116 subjects from the Karolinska COSMIC cohort (Table 1) matched for age and gender from the groups of healthy never-smokers, smokers with normal lung function, and COPD patients with mild to moderate disease (GOLD stage I-II/A-B; FEV1=51-97%; FEV1/FVC<70%).

Study participants were recruited from individuals performing spirometry during "The World Spirometry Day," through advertisements in the daily press and via primary care centers. The majority of the individuals with COPD were smokers who were found to have an obstructive spirometry upon screening. Participants had no history of allergy or asthma, did not use inhaled or oral corticosteroids and had no exacerbations for at least 3 months prior to study inclusion. In vitro screenings for the presence of specific IgE antibodies (Phadiatop; Pharmacia Corp) were negative. Reversibility was tested after inhalation of two doses of 0.25 mg terbutaline (Bricanyl; Turbuhaler®; AstraZeneca). Medications (including oral contraceptives, estrogen replacement and NSAIDs or other potential lipid mediator-modifying drugs) were recorded by means of a questionnaire. Lung function parameters were calculated as post-bronchodilator percent of predicted using the European Community of Coal and Steel (ECCS) normal values. COPD patients and smokers were matched in terms of smoking

history (>10 pack years) and current smoking habits (>10 cigarettes/day the past 6 months). Current smokers were asked to refrain from smoking at least 8 hr prior to sampling by venipuncture and bronchoalveolar lavage, which was confirmed by measurement of the level of exhaled carbon monoxide [5]. The COPD group consisted of both current smokers and ex-smokers (>2 years since smoking cessation). The current study was approved by the Stockholm Regional Ethical Board (COSMIC cohort: Case No. 2006/959-31/1) and participants provided their informed written consent.

Sample collection and preparation

Blood was drawn between 7-9 AM from fasting individuals by venipuncture and allowed to stand at room temperature for at least 30 min before centrifugation at 1695 ×*g* for 10 min at room temperature, and stored at -80°C until use. High sensitive C-reactive protein, platelets and leukocytes counts were measured according to standard methods at the Department of Clinical Chemistry, Karolinska University Hospital.

Airway epithelial brushings as well as bronchoalveolar lavage (BAL) samples were collected by means of fiberoptic bronchoscopy as previously described [6-8]. BAL samples were collected from the middle-lobe bronchus using 5×50 mL of phosphate buffered saline (PBS, 37°C). The combined aspirates were filtered and centrifuged at 400 *g* for 5 min at 4°C. Supernatant was isolated and 2 mL aliquots were kept in -80ºC until analysis.

Sample preparation and mass spectrometric analysis for non-targeted metabolomics

<u>Hydrophilic interaction liquid chromatography (HILIC) sample extraction:</u> On the day of analysis samples were thawed on ice. Serum protein was precipitated using 200 µL of HPLC grade acetonitrile (Rathburn Chemicals) in 50 µL of serum. Samples were vortexed for 5 sec, left to stand on ice for 10 min before centrifuging (Eppendorf Centrifuge 5430 R) at 15000 ×*g*

for 10 min at 4°C. 150 μL of supernatant were then transferred to a clean eppendorf tube and stored at -20°C until the day of analysis. A 50 μL aliquot of the HILIC extract was transferred to a Chromacol vial (03-FISV Thermo Fisher) on the day of analysis and capped with a pre-slit PFTE caps (03-FISV Thermo Fischer 9-SC(B)-ST1X). Quality control (QC) samples were prepared by pooling aliquots of 20 μL of each sample and an extraction blank was prepared by replacing serum with water (Milli-Q, Millipore). Both blank and QC samples were prepared along with the study samples following the same extraction procedure.

Reversed phase (RP) sample extraction: On the day of analysis, samples were thawed on ice and serum protein was precipitated using 150 μL of ice-cold (-20°C) HPLC grade methanol (Rathburn Chemicals) in 50 μL of serum. Samples were vortexed for 5 sec, left to stand on ice for 10 min before centrifuging (Eppendorf Centrifuge 5430 R) at 15000 ×*g* for 10 min at 4°C. Samples were vortexed for 5 sec, left to stand at -20°C for 2 hr before centrifuged (Eppendorf Centrifuge 5430 R) at 15000 ×*g* for 10 min at 4°C. The supernatant was transferred to a clean eppendorf tube and stored in -80°C until the day of analysis. On analysis day, 20 μL of the extract were diluted 1:1 with HPLC grade water (Milli-Q, Millipore) and transferred to a Chromacol vial (03-FISV Thermo Fischer) and capped with a pre-slit PFTE caps (03-FISV Thermo Fischer 9-SC(B)- ST1X). The QC samples were prepared by pooling aliquots of 20 μL of each sample, and an extraction blank was prepared, replacing serum with water (Milli-Q, Millipore). Both blank and QC samples were prepared along with the study samples following the same extraction procedure.

HILIC LC-HRMS analysis: Study samples, QC, blanks and external identification standard mixtures were analyzed on a Thermo Ultimate 3000 UHPLC and Thermo Q-Exactive Orbitrap mass spectrometer as previously described [9]. 12 μL of sample were injected on a

Merck-Sequant ZIC-HILIC column (150×4.6 mm, 5 µm particle size) fitted with a Merck Sequant ZIC-HILIC guard column (20×2.1 mm). A 40 min gradient (0.3 mL/min flow rate, 23°C column oven) using 0.1% formic acid in HPLC water (mobile phase A) (Milli-Q, Millipore) and 0.1% formic acid in HPLC acetonitrile (mobile phase B) (Rathburn Chemicals) was applied. The gradient started at 80% B, reducing to 20% B after 30 min, followed by immediate return to initial conditions and a 10 min column re-equilibration. Mass spectrometry data were acquired (full scan mode) in both positive and negative ionization modes (an independent run for each polarity), using a mass range of 75 and 1000 *m/z* with a resolution of 140,000 at 400 *m/z*. In positive mode, the spray voltage was 4.0 kV with a capillary temperature of 350°C, a sheath gas flow of 30 and an auxiliary gas flow of 10 (arbitrary units by vendor). In negative mode, the spray voltage was 3.6 kV with a capillary temperature of 350°C, a sheath gas flow of 30 and an auxiliary gas flow of 12 (arbitrary units by vendor). Samples were randomized across the whole sequence to prevent potential confounding signal drift. Five laboratory reference serum samples were used at the beginning of each sequence for conditioning and every six randomized clinical samples were bracketed by a QC sample.

<u>RP LC-HRMS analysis:</u> Study samples, QC and blanks were analyzed on a Thermo ultimate 3000 HPLC and Thermo Q-Exactive Orbitrap mass spectrometer as previously described [9]. 20 µL of samples were injected on a Thermo Accucore aQ RP C18 column (150 × 2.1 mm, 2.7 µm particle size). A 27 min gradient was used with a flow rate of 0.65 mL/min, a column temperature of 40°C, 0.1% formic acid in HPLC water (Milli-Q, Millipore) as mobile phase A and 0.1% formic acid in HPLC acetonitrile (Rathburn Chemicals) as mobile phase B. The gradient started with a 3 min isocratic flow 0.1% B, followed by a linear increase to 99.9% B at 19 min, then a 3 min isocratic flow and a rapid restoration of starting conditions and

column re-equilibration for 5 min. The flow was split post-column 1:1 (source:waste) using an adjustable flow splitter (Scantec Lab, AB, Gothenburg). Mass spectrometry data were acquired (full scan mode) in both positive and negative ionization modes, an independent run for each polarity, using a mass range of 130-900 with 70,000 mass resolution at 400 *m/z*. In positive mode, the spray voltage was 4.0 kV with a capillary temperature of 350°C, a sheath gas flow of 30 and an auxiliary gas flow of 10 (arbitrary units by vendor). In negative mode the spray voltage was 3.6 kV, with a capillary temperature of 350°C, a sheath gas flow of 40 and an auxiliary gas flow of 12 (arbitrary units by vendor). Samples were randomized across the whole sequence to prevent potential confounding signal drift. Five laboratory reference serum samples were used at the beginning of each sequence for conditioning and every six randomized clinical samples were bracketed by a QC sample.

Data Processing

The raw files were converted to mzXML and centroid using MSconvert. All chromatograms were evaluated using the open source software package XCMS performed under the package R. The mzXML files were organized in the working directory by sample class. Peak picking was performed using the centwave method, allowing the de-convolution of closely eluting or partially overlapping peaks. The peak width range parameter was set to (3-25 sec) for RP and (10-50 sec) for HILIC.

Putative Metabolite Annotation Method

Metabolite suggestions for significant metabolite peaks were identified by initially querying the *m/z* values using the Human Metabolome Database's MS search functionality with the "molecular weight tolerance" set to 0.001 Da for HILIC peaks and 0.005 Da for RP peaks [10]. If these settings failed to yield an annotation, a broader search was performed in both the

Kyoto Encyclopedia of Genes and Genomes (KEGG) and Lipid Maps databases using the "putative ionization product" interface of the annotation tool MZedDB (http://maltese.dbs.aber.ac.uk:8888/hrmet/search/addsearch0.php) using a mass accuracy of 20 ppm for both HILIC and RP. Once metabolite suggestions had been generated, annotation was performed in two steps. Where possible, the first step was to try to determine the molecular formula of the underlying metabolite matching the isotope ratio observed for the annotated peak, and the 6 expected isotope ratios for the suggested metabolites. The second step aimed to narrow the metabolite suggestions to a single metabolite annotation. For HILIC and RP peaks, published literature and metabolite databases were interrogated, to ascertain whether metabolite suggestions had been reported in blood or other human biofluids, and if the suggested metabolite ID was plausible within the given biological context.

Metabolite annotation with in-house library

Metabolic features from the XCMS output were matched to an in-house accurate mass/retention time library of reference standards to increase the accuracy of the metabolite annotation. The library consisted of several compound classes ranging from polar to non-polar (*e.g.,* carboxylic acids, amino acids, biogenic amines, polyamines, nucleotides, vitamins and coenzymes, sugars, carnitines, fatty acids, phospholipids, sphingolipids, ceramides and steroids) [9]. Metabolites were annotated by matching the accurate mass in HILIC (+/-0.0005 Da) and in RP (+/- 0.004 Da) and retention time (+/- 30 sec in HILIC and +/-10 sec in RP) of analyte peaks.

Metabolite selection and statistical analysis

The annotated metabolites from both putative and accurate mass/retention time (irrespective of HILIC and RP analyses) were combined into a single file to perform metabolite selection

and statistics. Four samples were not analyzed in HILIC positive mode due to lack of material. For the purpose of the statistical analysis, the corresponding missing values were imputed using k-nearest neighbors imputation [11]. The chromatographic signal drift (if any) was normalized with a QC normalization algorithm in MATLAB vR2015a (Mathworks, Natick, MA, USA) [12]. Statistical analysis was applied only to those metabolites that were present in ≥70% of the samples in any group and had a coefficient of variance <30% in the QC samples.

Univariate statistics was performed on the filtered data using the non-parametric Mann-Whitney test, and Storey's q-values were estimated using MATLAB vR2015a (Mathworks, Natick, MA, USA). Because age and smoking packyears differed significantly between the healthy smokers and COPD groups (Table E1), p-values were adjusted correspondingly using STATA v12 (StataCorp, Texas, USA).

SIMCA v14.0 (MKS, Sweden) was used on the filtered data for multivariate statistical analysis. The missing values (below the method detection limit) were replaced with the 1/3 of the lowest intensity for each corresponding metabolite. Data were log transformed and pareto scaled. Principal component analysis and orthogonal projections to latent structures with discriminant analysis (OPLS-DA) were performed on the filtered metabolites. An OPLS-DA model was built for each comparison (Smokers *vs.* COPD, female Smokers *vs.* COPD, and male Smokers *vs.* COPD) in order to identify metabolites that differed between the healthy individuals and COPD populations. Variable selection was performed on the initial OPLS-DA models in order to identify those metabolites that exhibited the strongest association with COPD. As previously described, metabolites with p(corr) values (the scaled loadings of the predictive component of the OPLS-DA model) ≥0.4 and variable importance in projection (VIP) values ≥1.0 were selected and used to generate new OPLS-DA models [13]. This process was performed iteratively, with performance monitored using the 7-fold cross-

validated ANOVA *p*-value (CV-ANOVA) [14]. If the CV-ANOVA *p*-value decreased, then the variable selection step was regarded as beneficial; however, if the *p*-value increased, then the variable selection round was rejected. The final OPLS-DA models were constructed using the MS/MS and/or standard confirmed metabolites (described below).

Metabolite confirmation

Metabolites that were significant via univariate analysis (Mann Whitney test $p<0.05$) were combined with the metabolites from multivariate analysis ($|p[\text{corr}]| \geq 0.4$ and VIP≥1.0) to generate a single list of metabolites for confirmation. An MS/MS experiment was performed on all selected metabolites (univariate and multivariate) with three different collision energy (15 eV, 35 eV and 50 eV) applying the same LC-HRMS conditions. Simultaneously the corresponding analytical standards (when available) were injected to confirm the accurate mass/retention time and MS/MS.

Targeted metabolomics using Biocrates Kit

Targeted metabolite quantitation was performed using the Biocrates AbsoluteIDQ p180 kit (Biocrates Life Sciences AG, Austria). The experiment was performed according to the Biocrates instructions for a Waters Xevo TQS triple quadrupole (direct flow injection analysis and LC-MS/MS) for the quantitation of 188 metabolites including amino acids, acylcarnitines, sphingomyelins, phosphatidylcholines, hexose (glucose) and biogenic amines. A full list of metabolites is available on the manufacturer's homepage (http://www.biocrates.com/products/research-products/absoluteidq-p180-kit). For the direct flow injection analysis, a 10 µL loop was used instead of the 20 µL loop recommended by the manufacturer. The assay was based on phenylisothiocyanate-derivatization in the presence of isotopically labeled internal standards followed by direct flow injection analysis tandem mass

spectrometry (acylcarnitines, lipids, and hexose) as well as LC-MS/MS (amino acids and biogenic amines). Multiple reaction monitoring (MRM) detection was used for quantitation. Concentrations of all analyzed metabolites were reported in μM. An HSS T3 (2.1×100 mm, 1.8 μm) column was used. The gradient and flow rate were adjusted according to the column length and the retention time for each metabolite using the test mixtures supplied by the manufacturer. Briefly, the flow rate decreased to 0.6 mL/min, the gradient started with 100% mobile phase A (0.2% formic acid in water), decreasing to 85% A to 2.5 min, followed by another linear decrease to 30% A to 5 min, reaching 100% B (0.2% formic acid in acetonitrile) at 5.30 min. From 5.30 to 7.00 min the gradient remained isocratic with 100% B. At 7.10 min the gradient was returned to initial conditions and maintained until 8.50 min. MS conditions were as recommended by the manufacturer. Both univariate (non-parametric Mann-Whitney test and Storey's q-value) and multivariate statistical analysis (SIMCA v14.0) were performed on the targeted profiling data sets using the methods as described above.

MiRNA profiling

As described previously [15], RNA was extracted and separated into small RNA (including miRNAs, 18-200 nt) and large RNA (>200 nt) fractions by using Nucleospin miRNA, according to the manufacturer's instructions. RNA quality was assessed by using UV 260/280 and 230/260 absorbance ratios obtained by using Nanodrop (Thermo Scientific, Wilmington, DE), resulting in a mean 260/280 ratio of 1.95. RNA size distribution was examined on RNA Pico LabChips (Agilent Technologies, Palo Alto, CA) processed on the Agilent 2100 Bioanalyzer small RNA electrophoresis program. An aliquot of 1 mL was used for validation by means of quantitative RT-PCR, and the rest was concentrated (SpeedVac, Thermo Fisher) to a volume of 4 mL and used for amplification. RNA was labeled with Cy3-CTP by using the miRCURY LNA microRNA power labeling kit (Exiqon, Woburn, MA), according to the

manufacturer's protocol. Labeled RNA was hybridized to 1-color Agilent custom UCSF miRNA v3.5 multi-species 8x15K Ink-jet arrays (Agilent Technologies) containing 894 different miRNAs.

miRNA from BAL cells, BEC, and exosomes from BAL fluid were only analyzed from a subset of the Karolinska COSMIC cohort based upon sample availability (n=45; 5-13 subjects per group and gender). For the BAL cells, sub-group sizes were n=9-13, and thereby represent approximately half of the total study. The potential for selection bias was evaluated by examining the distribution of the subjects in PCA scores plot based on metabolic profiles or clinical data (data not shown). Based upon these parameters, it was determined that there was no selection bias in the measured sub-cohort. For the BEC cells, sub-group sizes were n=5-13. Due to the smaller sample numbers in the BEC subgroups, the correlation analyses presented in the study were only performed for the BAL cells. As evident from Figure E6, the large difference in miRNA levels between female and male smokers is apparent in spite of the relatively small subgroup sizes.

Brief tutorial on multivariate projection methods used in this study:

Overview

Multivariate statistical modelling methods are a set of tools aimed at reducing the dimensionality of the complex, multidimensional data structures encountered within metabolomics and other 'omics-based disciplines [16, 17]. The common denominator is that these experimental platforms generate so-called "short-and-wide" data tables with a large number of variables (hundreds to thousands) measure in a limited number of subjects. These types of data sets are recalcitrant to analysis with standard univariate approaches. As a complement to standard univariate statistical methods, these high dimensional data can be analysed and interpreted by multivariate models. There are multiple multivariate methods available, and it is beyond the scope of the text here to expand upon them all. Interested readers are directed to basic texts [18]. Instead, we will focus on the methods used in the current study: Principal component analysis (PCA) [19], partial least-squares to latent structures (PLS) [20, 21] and orthogonal projections to latent structures (OPLS) [22-24]. Collectively, these approaches include efficient and robust methods for analysis, and visualization of complex chemical and biological data.

Principal component analysis (PCA)

Principal component analysis forms the basis for multivariate data analysis. The starting point for PCA is a matrix of data with N rows (*observations, here study subjects from which the serum samples have been collected*) and K columns (*variables, here metabolites*), often referred to as the X-matrix. The most important use of PCA is to represent a multivariate data table as a low-dimensional plane, usually consisting of 2 to 5 dimensions, which provides an overview of the data. This approach quickly reduces a high-dimensional dataset to a low-dimensional plane consisting of a few latent variables that can be more easily comprehended.

This overview may reveal groups of observations, trends, and outliers, serving as a useful data quality control step. This overview also uncovers the relationships between observations, which are displayed in the *scores plot*, and variables, which are displayed in the *loadings plot*.

Scaling

Prior to multivariate modeling, data are often pre-treated in order to transform the data into a form suitable for analysis. In metabolomics, the variables (metabolites) often have substantially different dynamic concentration ranges. A metabolite (variable) with a large range has a large variance, whereas a metabolite (variable) with a small range has a small variance. Since PCA is a maximum variance projection method, it follows that a metabolite (variable) with a large variance will drive the model compared to a low-variance metabolite (variable) similar to how a high concentration sample will drive the correlation curve in a standard curve for e.g., protein quantification methods The most common form of pre-treatment is mean centering and scaling. In mean centering, the average value of each metabolite across all subjects is calculated and then subtracted from that of each individual value. In terms of scaling, the most common technique in univariate modeling is the *unit variance (UV) scaling,* sometimes referred to as *auto-scaling*. Following mean centering, each individual metabolite value is divided by the standard deviation (*SD*) for the metabolite across all subjects in the study. Subsequently, each scaled variable then has equal (unit) variance. Taken together, mean centering and scaling to unit variance removes the influence of metabolite abundance in the model, so that alterations of low abundance metabolites are equally important as alterations in high abundance metabolites, which represents a biologically more valid approach.

PCA model calculations

Consider again the X-matrix with *N* samples and *K* metabolites. For this matrix, we construct a metabolic space with as many dimensions as there are metabolites. Each metabolite represents one co-ordinate axis. For each metabolite, the length has been standardized according to the chosen scaling criterion, normally by scaling to unit variance. In the next step, each sample (each row) of the X-matrix is placed in the *K*-dimensional metabolic space. Consequently, the samples in the data table together form a swarm of points in this space as the metabolite concentrations for each sample (row) make up the coordinates in this *K*-dimensional metabolic space. The mean-centering of the data corresponds to a re-positioning of the swarm of points to the origin.

After mean-centering and UV scaling, the dataset is ready for the computation of the first latent variable, or principal component (PC1). This component is the line that represents the largest variance in the *K*-dimensional metabolic space. This line goes through the origin (average metabolic profile). Each sample (point in this *K*-dimensional space) is thereafter projected onto this principal component line, which becomes its co-ordinate value along this PC-line. This co-ordinate value for each sample is known as a *score*, and collectively for all samples they are termed *scores*. Usually, one principal component line is insufficient to model the systematic variation of a multi-dimensional metabolite dataset, and a second principal component, PC2, which is orthogonal (perpendicular) to PC1, is calculated. This line also passes through the average point, and improves the approximation of the X-data as much as possible. Again, each sample is projected onto this second principal component line, and its co-ordinate value is the second score value, generating a second set of scores for all of the metabolites.

When two principal components have been derived, they together define a plane, or a "cross-section" of the *K*-dimensional metabolite space. The projection of all the samples onto

this two-dimensional sub-space is the scores values, and by plotting the results as a two-dimensional scatter plot (PC1 *vs.* PC2), it is possible to visualize the structure of the metabolite dataset. The co-ordinate values of the samples (*scores)* are plotted and the plot is therefore known as a *scores plot*. The scores plot provides information on the individual clinical samples (*e.g.,* patients); however, there is a corresponding plot for the metabolites called the *loadings plot*. This plot reveals how the metabolites contribute to the structure of the scores plot, and is essential for model interpretation. The loadings plot can be used to link information between individual variables (*e.g.,* metabolites) and clinical samples, for example, it can help understand which metabolites are driving an observed separation of samples in the scores plots. In a similar fashion, a second set of loading coefficients expresses the direction of PC2 in relation to the original variables. The residual matrix E contains the residuals for each sample between its point in *K*-dimensional space and its point on the model plane. The residuals are important for detection of outliers and for defining the model boundaries.

Orthogonal Projections to Latent Structures-Discriminant Analysis (OPLS-DA)

In contrast to the more commonly used PCA modeling, orthogonal projections to latent structures (OPLS) analysis is a supervised method designed to separate structured noise unrelated (orthogonal) to the predictive variance of interest (*e.g.,* Healthy *vs.* COPD). [25]. In its simplest form, OPLS is used as a discriminant analysis (OPLS-DA) method to evaluate the ability to classify known groups of subjects (*e.g.,* Smokers with normal lung function *vs.* COPD patients). This additional class information is defined in a second data set, the Y-matrix. The filtering of the variation in the X-data, of the within-class variance (also known as orthogonal or uncorrelated variation) from the class-separating variance (also known as predictive variation) greatly increases the interpretability of the multivariate model,

particularly in terms of deriving the observed group separation back to the variables (metabolites) of interest. In addition, the OPLS method can also be used to predict the class belonging of new unknown samples. The predictive power can be estimated through cross-validation (please see below).

Model statistics

For PCA and OPLS-DA models, the amount of modeled variation is defined as the goodness of fit (R^2), where an R^2 value of 1.0 indicates that all variation in the data is modeled, and a value of 0.0 means that no variation in the data is modeled:

Goodness of fit: $R^2(\mathbf{Y}) = 1 – SS(\mathbf{F})/SS(\mathbf{Y})$

However, as R^2 only relates to the goodness of fit for the dataset at hand, a goodness of prediction (Q^2) is also reported. The Q^2 is calculated is the based on cross validation [26]; a subset of the subjects are left out and a new classification model is constructed based on the remaining subjects. The group belonging to the excluded subjects is then predicted based on the new model. This process is repeated until all subsets have been excluded and predicted. Q^2 values of 1.0 reflect perfect predictive precision, while values equal to or below 0.0 indicate that a random guess is more accurate than the model's own predictions:

Goodness of prediction: $Q^2(\mathbf{Y}) = 1 - PRESS/SS(\mathbf{Y})$

PRESS: predictive error sum of squares

For supervised approaches such as OPLS-DA, analysis of variance analysis (ANOVA) formally compares two (or several) models fitted to the same data by the size of their fitted residuals. ANOVA is made on the size of the sum of squares (SS(d)) and (SS(e)), and uses an F-test for the significance test (hypothesis test) of the null hypothesis of equal residuals of the two models. The F-tests assume that the residuals of the two compared models are approximately normally distributed. The corresponding mean squares (MS), or variances, are

obtained by dividing each SS by the respective degrees of freedom. The F-test, based on the ratio MS regression/MS residual, then formally assesses the significance of the model. The p-value indicates the probability level for a model with this F-value being the result of just chance.

Herein, we have used a recent extension, CV-ANOVA [14], based on cross-validated predictive residuals to provide a significance metric for multivariate regression models, including OPLS-DA models. The CV-ANOVA diagnostic corresponds to a hypothesis test of the null hypothesis of equal cross-validated predictive residuals of the two compared models. Naes and co-workers [27] have shown that cross-validated residuals are relevant and work well in the context of ANOVA, and are more reliable than ordinary ANOVA. This is particularly important in multivariate OPLS-DA models where the number of X-variables is often large.

Group classification and biomarker selection using OPLS

One of the major strengths of supervised methods, particularly OPLS, is its application in variable selection. Variable selection is an essential step in identifying and evaluating the performance of subsets of variables for classification of patient subgroups (*e.g.,* biomarker discovery). In MVA the question of which variables are of interest, corresponding to determining significance in univariate statistics, is not trivial. General rules for where to apply the cutoff in the continuous variable ranking, such as $p<0.05$ in univariate statistics, have not yet been established. The use of a Variable Influence on Projection (VIP; also referred to as Variable Importance in Projection) score>1.0 is common in publications. VIP is a metric that summarizes the importance of each variable in driving the observed group separation [28]. However, VIP>1.0 only implies that the variable contributes more than average to the model, and the VIP>1.0 cutoff results in selection of up to 50% of the variables. In addition, the VIP

score is a relative ranking term that changes with each iteration of variable selection, rendering it somewhat of a moving target. It is therefore often difficult to determine the optimal model based solely upon VIP values. An alternative and complementary parameter is the p(corr) value. P(corr) is the loadings scaled as a correlation coefficient, thereby standardizing the range from -1.0 to 1.0. The p(corr) values remain stable during iterative variable selection and are comparable between models. There is no consensus on what p(corr) cutoff represents significance, but an absolute p(corr)>0.4-0.5 is often used [29-33]. For variable selection, we recommend the use of a combination of p(corr) and VIP. A constant p(corr) can be used as a cutoff point for variable selection if the aim is to maximize the statistical power. Alternatively, if the goal is to select a subset of biomarkers, several iterations of variable selections can be performed as long as the Q^2 and CV-ANOVA p-value continue to increase.

Overfitting is an inherent risk in OPLS analysis, and determining the appropriate number of components is essential, but not always trivial. The default automatic fitting in SIMCA extracts the maximal number of significant components, which in most cases results in an overfitted model. The result is an inflated R^2, but a lowered Q^2 because the overfitting occurs at the expense of the predictive power. The optimal number of components is at the break point where Q^2 decreases with the addition of more components. The CV-ANOVA p-value can be used as a complement to the Q^2 for determining the optimal number of components (an increasing p-value due to addition of a component implies overfitting).

Summary

Technical aspects of the OPLS algorithm (the basis for OPLS-DA) have been described fully by Trygg and Wold [22]. A technical description of OPLS-DA, together with application studies, has been provided by Bylesjö et al. [34]. Examples of the application of multivariate

statistical analysis (MVA) to ‘omics-based analyses in respiratory disease can be found for asthma [30-32, 35-37], COPD [4, 29, 38-44], pulmonary hypertension [45] and sarcoidosis [46]. For a description of the challenges and necessary metrics in interpreting multivariate models, interested readers are referred to a number of recent papers [17, 47, 48]. It is anticipated that the use and application of these multivariate modeling approaches will continue to increase in ‘omics based science in biomedicine. It is therefore important that the biomedical research community becomes familiar with these statistical approaches.

Supplementary Figures

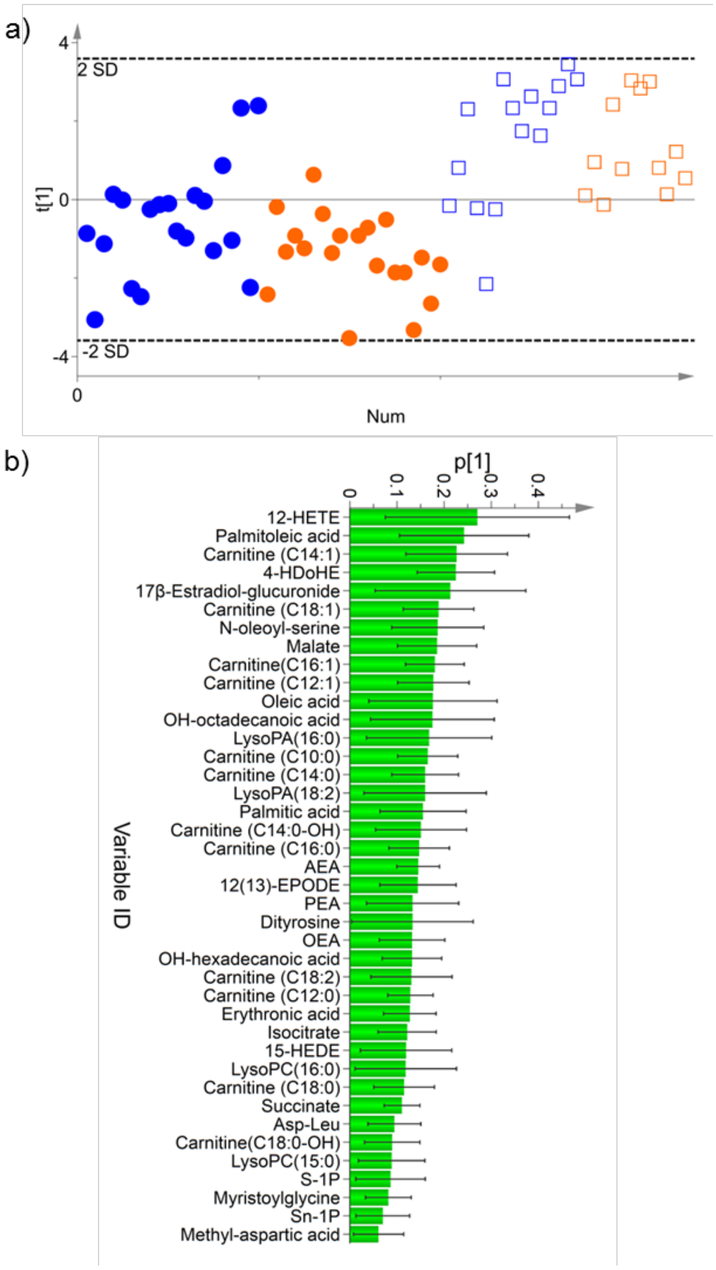

Figure E1: Joint gender multivariate model for Smokers *vs.* COPD. a) OPLS-DA scores plot for Smokers *vs.* COPD groups (n=58 metabolites, $R^2Y= 0.45$, $Q^2= 0.38$, $p=2.8\times10^{-7}$) with the predictive component along the *y-axis*. Because no orthogonal components were required, the *x-axis* merely represents a numeric ordering (Num) of the samples (open box, individuals with COPD; closed circle, Smokers; blue symbols indicates male and orange symbols indicate females). The receiver operating characteristic (ROC) curve for classification of smokers with normal lung function from smokers with COPD had an AUC=0.90; b) Loadings plot of verified metabolites prominent for driving the separation between Smokers *vs.* COPD.

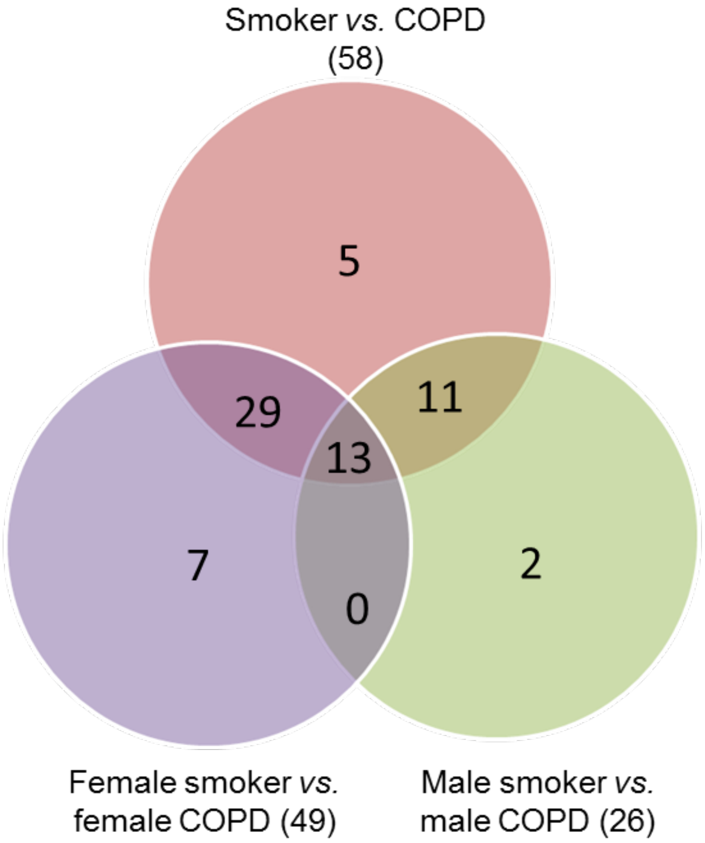

OH-hexadecanoic acid	Erythronic acid
Carnitine (C14:0)	Carnitine (C14:1)
Carnitine (C16:1)	Carnitine (C18:1)
Carnitine (C18:2)	Malate
Methylhippurate	12-HETE
Succinate	4-HDoHE
LysoPA(16:0)	

The panel displays the 13 metabolites common to all groups.

Figure E2: Venn diagram, showing overlap and unique metabolite distribution for the joint gender (Smokers *vs.* COPD, n=58 metabolites, $R^2Y=0.45$, $Q^2=0.38$, $p=2.8\times10^{-7}$), male gender (male Smokers *vs.* male COPD, n=26 metabolites, $R^2Y=0.49$, $Q^2=0.38$, $p= 4.0\times10^{-4}$) and female gender (female Smokers *vs.* female COPD, n=49 metabolites, $R^2Y=0.73$, $Q^2=0.65$, $p=2.4\times10^{-7}$).

Definition of abbreviation: 12-HETE = 12-Hydroxyicosatetraenoic acid, 4-HDoHE = 4-Hydroxydocosahexaenoic acid, OH = Hydroxy, LysoPA= Lysophosphatidic acid.

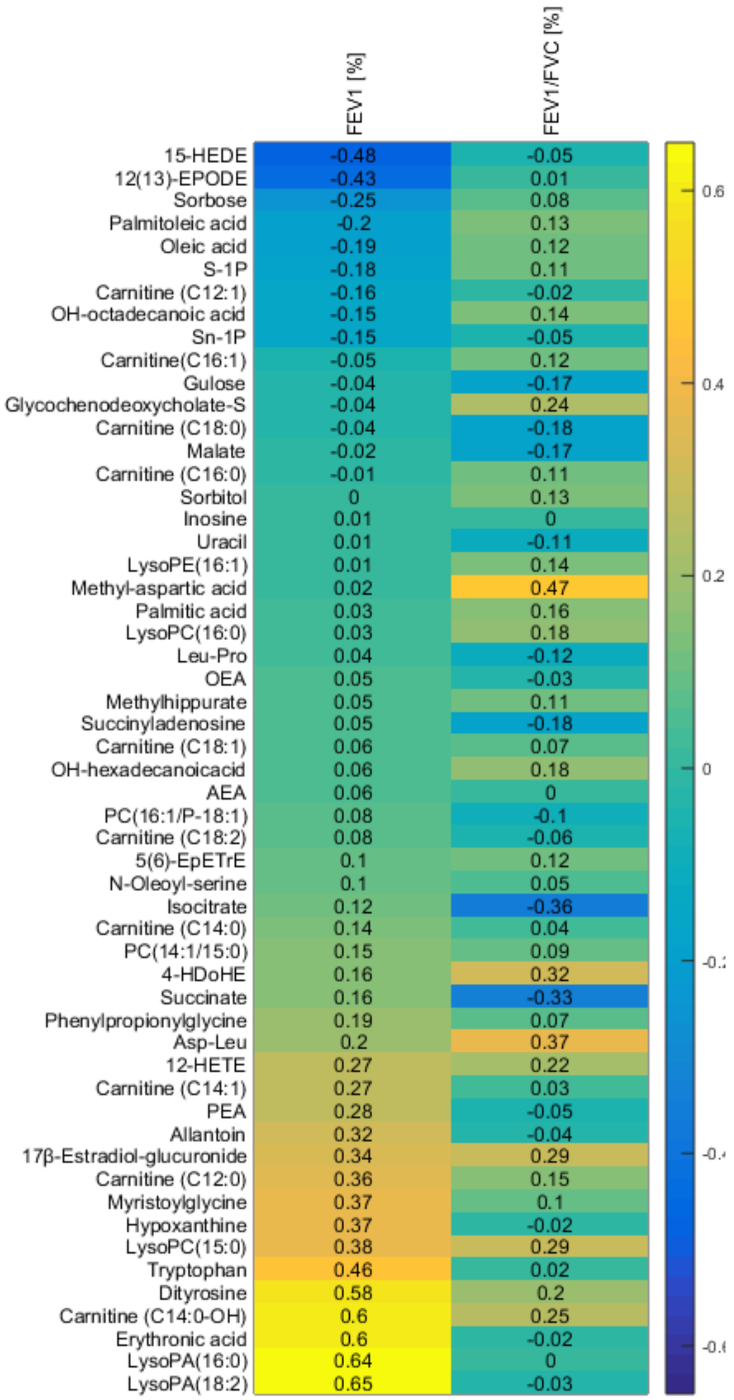

Figure E3: A heat map generated using the spearman rank correlation *rho* value. The correlation was performed between lung parameters (FEV_1 (%) predicted and FEV_1/FVC (%)) and 58 significant circulating metabolites with altered levels due to COPD. Definition of abbreviations: 12(13)EpODE = 12(13)-Epoxyoctadecadienoic acid, 12-HETE = 12-Hydroxyicosatetraenoic acid, 15-HEDE =15-Hydroxyeicosadienoic acid, 4-HDoHE = 4-Hydroxydocosahexaenoic acid, 5(6)-EpETrE = 5(6)-Epoxyeicosatrienoic acid, AEA = *N*-arachidonoylethanolamine, Asp-Leu = Aspartic acid-Leucine, Leu-Pro = Leucine-Proline, LysoPA = Lyso-phosphatidic acid. LysoPC = Lysophosphatidylcholine, LysoPE = Lysophosphatidylethanolamine, OEA = *N*-oleoylethanolamine, PC = Phosphatidylcholine, PE = Phosphatidylethanolamine, PEA = *N*-palmitoylethanolamide, S-1P = Sphingosine-1-phosphate, Sn-1P = Sphinganine-1-phosphate.

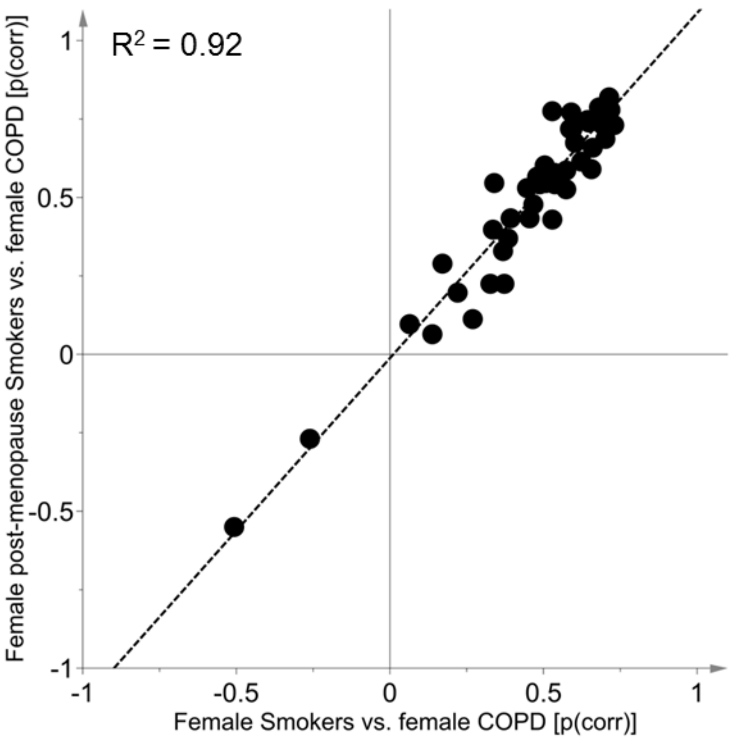

Figure E4: Shared and Unique Structures (SUS) analysis examining the effects of menopausal status upon observed COPD-associated effects on metabolite levels. A SUS plot displays the correlation between two OPLS models, displaying whether any metabolites have behavior that is unique for one of the OPLS models, or if metabolites behave the same (shared) in both models (15). The closer the metabolite distribution is to a perfect diagonal (R^2=1.0), the more shared structure in the models. This figure compares the OPLS models between all female Smokers *vs.* females with COPD (*x-axis*, *n*=49 metabolites, R^2Y= 0.73, Q^2= 0.65, *p*=2.4 $\times 10^{-7}$) and female postmenopausal Smokers *vs.* female postmenopausal individuals with COPD (*y-axis*, *n*=49 metabolites, R^2Y= 0.75, Q^2= 0.67, *p*=9.6 $\times 10^{-6}$). The strong diagonal distribution of the metabolites indicates that all metabolites are behaving similarly in both models, thus demonstrating that there is no unique behavior for pre- *vs.* postmenopausal individuals with COPD. Models were generated using the non-targeted metabolomics data.

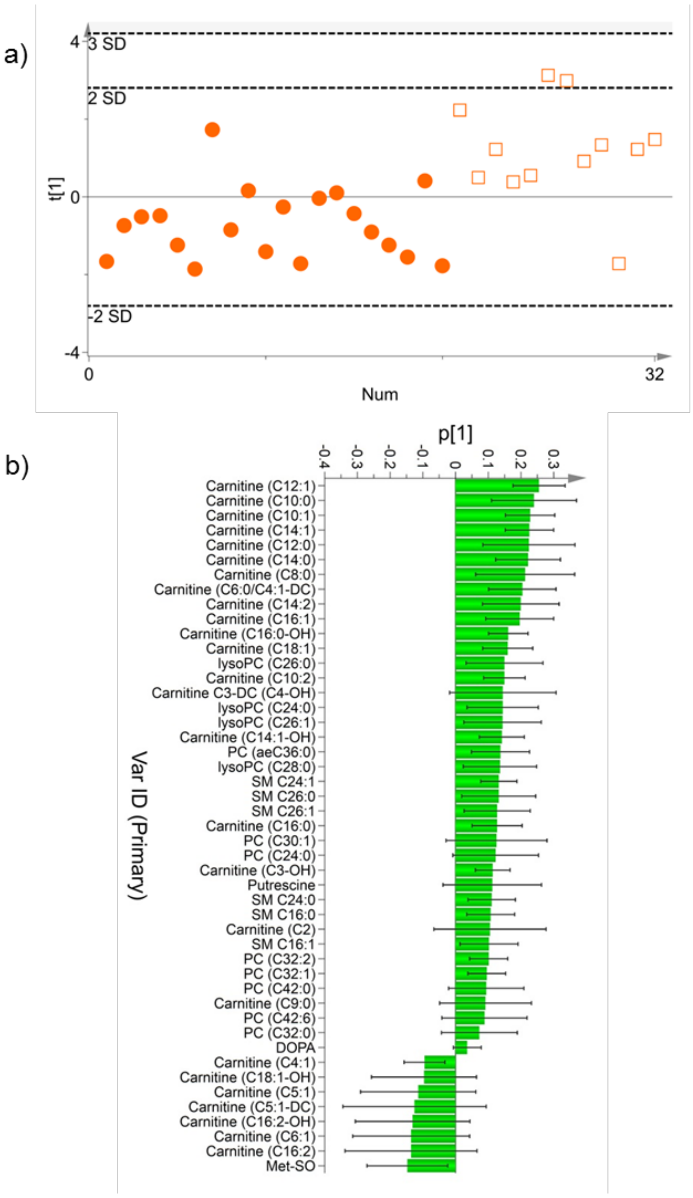

Figure E5: Optimized multivariate model using metabolites from the targeted metabolomics platform (Biocrates kit). a) Optimized OPLS-DA model for female Smokers *vs.* female COPD patients (metabolites=47, $R^2Y= 0.45$, $Q^2= 0.34$, p=0.003, filled circle = Smokers and open box = COPD individuals) with the predictive component along the *y-axis*. Because no orthogonal components were required, the *x-axis* merely represent a numeric ordering (Num) of the samples and the receiver operating curve AUC=0.89. b) Loadings of confirmed metabolites prominent for driving the separation between female Smokers vs. female COPD. The optimized model parameters for male Smokers *vs.* male COPD comparison for 54 metabolites are $R^2Y= 0.38$, $Q^2= 0.11$, p=0.1.

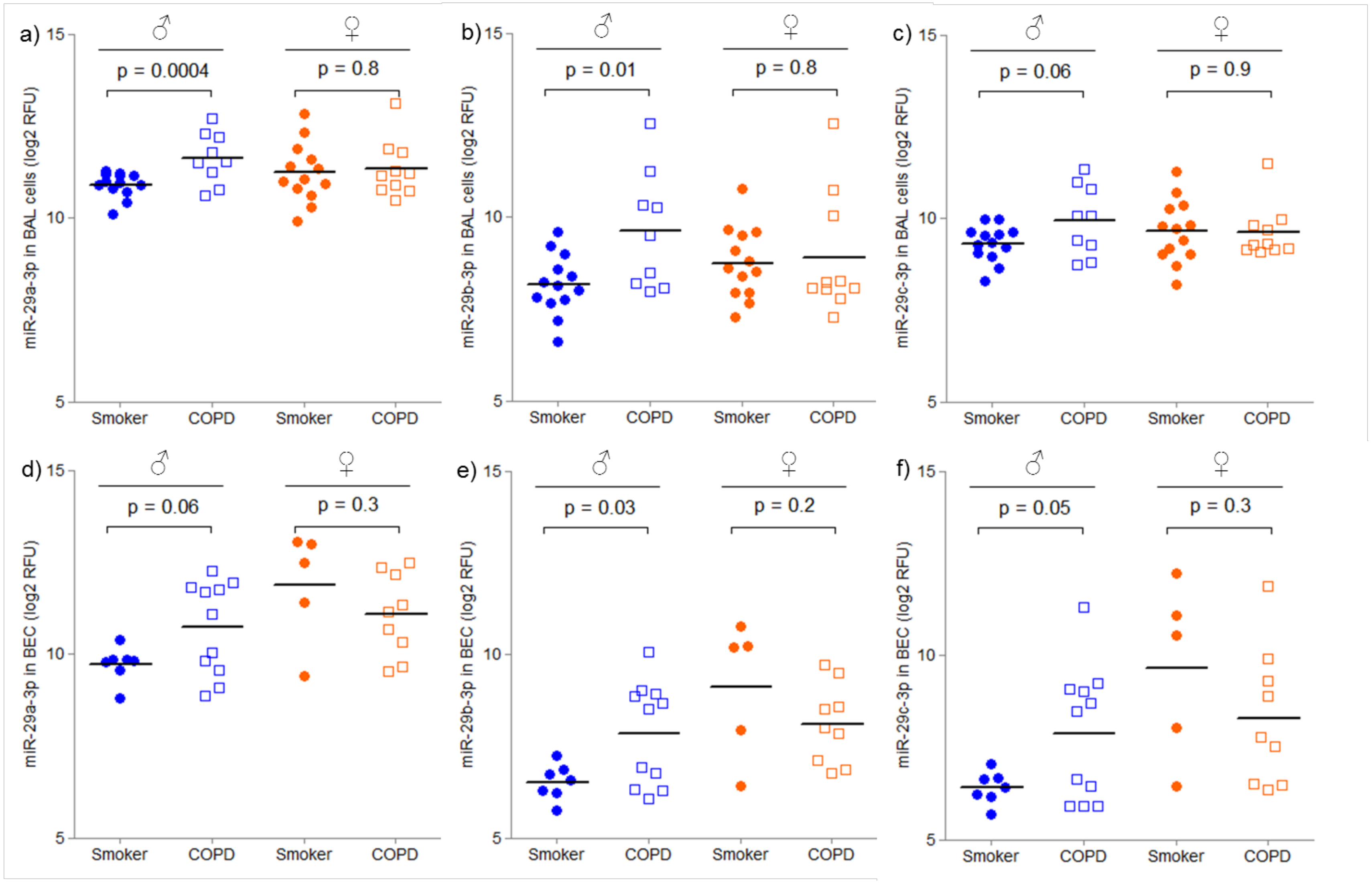

Figure E6. Levels of the miR-29 family in BAL cells and BEC from male and female Smokers and individuals with COPD. Subjects are divided into smokers with normal lung function (Smokers, filled circles) and smokers with COPD (COPD, open boxes). Blue symbols indicate males and orange symbols females. Significance is indicated by the non-parametric Mann-Whitney test.

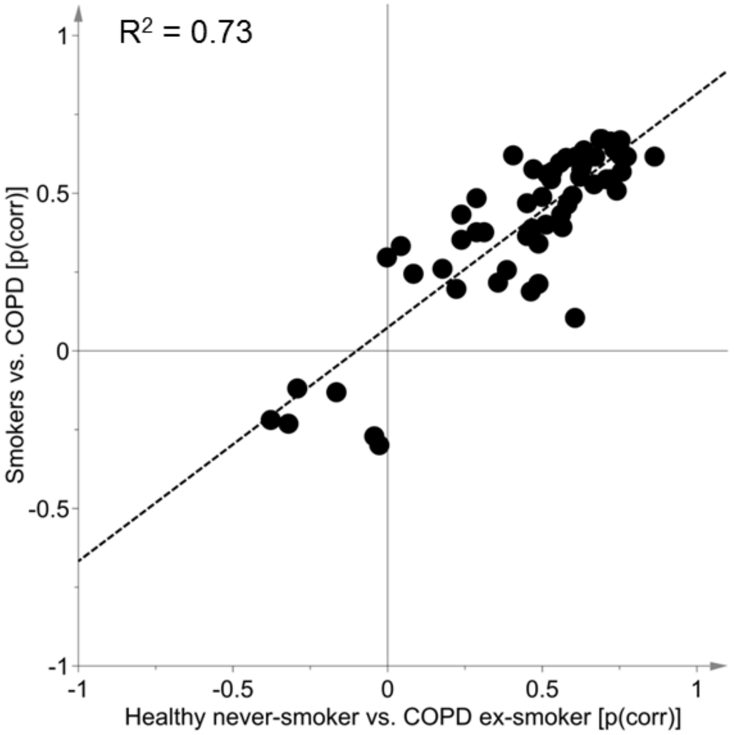

Figure E7: Shared and Unique Structures (SUS) analysis examining the COPD-specific effects upon metabolite levels. A SUS plot displays the correlation between two OPLS models, displaying whether any metabolites have behavior that is unique for one of the OPLS models, or if metabolites behave the same (shared) in both models (15). The closer the metabolite distribution is to a perfect diagonal (R^2=1.0), the more shared structure in the models. This figure compares the OPLS-DA joint gender models of Healthy *vs.* COPD-ExS (*x-axis*, *n*=58 metabolites, R^2Y= 0.17, Q^2= 0.04, *p* =4.0 ×10-2) and Smokers *vs.* COPD (*y-axis*, *n*=58 metabolites, R^2Y= 0.45, Q^2= 0.38, *p*=2.8 ×10-7). Although the small number of Ex-smoker COPD patients included in the study resulted in limited power for the non-smoker model, the tight clustering around the diagonal indicates that the same metabolites are altered due to COPD, regardless of current smoking status. Models were generated using the non-targeted metabolomics data.

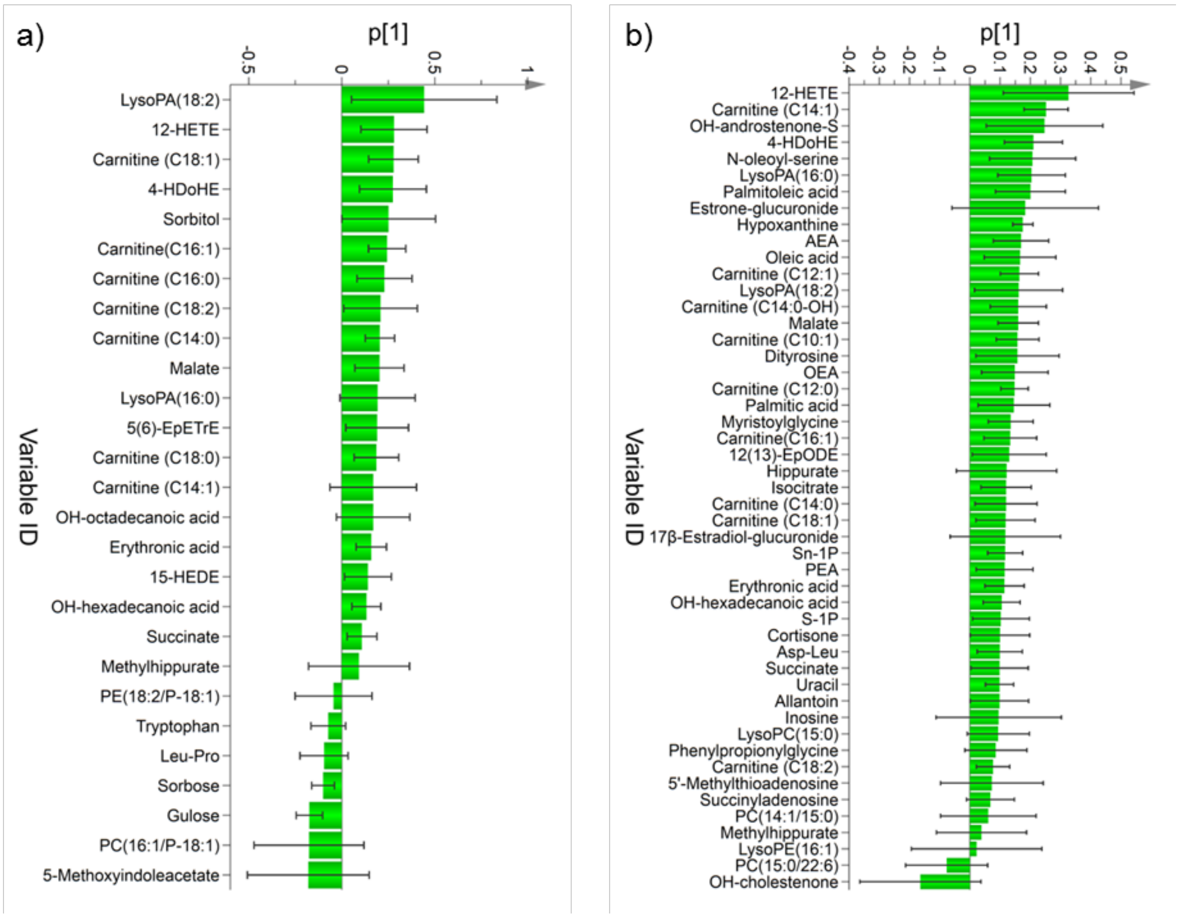

Figure E8. Loadings plot displaying all selected variables from the optimized multivariate models using non-targeted metabolomics from Figure 1. a) Loadings of verified metabolites most prominent for driving the separation between male Smokers *vs.* male COPD; b) Loadings of the verified metabolites most prominent for driving the separation of female Smokers *vs.* females with COPD.

Table E1: Significance analysis on the clinical parameters of subjects included in statistical analyses.

Parameters	Smoker *vs.* COPD			Healthy *vs.* COPD Ex-S
	♀♂	♀	♂	
Age	3.0×10^{-5}	8.0×10^{-4}	1.0×10^{-3}	0.1
BMI	1.0	0.7	0.8	0.7
Smoking (packyears)	2.0×10^{-3}	0.1	0.1	N.A.
Blood leucocytes ($x10^9$/L)	0.2	0.2	0.9	0.04
Blood platelets ($x10^9$/L)	0.3	0.8	0.1	1.0
Serum albumin (g/L)	0.2	0.4	1.0×10^{-4}	0.5
Serum antitrypsin (g/L)	4.0×10^{-3}	0.4	5.0×10^{-3}	0.6
FEV_1 (%)	1.7×10^{-14}	3.7×10^{-6}	1.5×10^{-9}	2.7×10^{-7}
FEV_1/FVC (%)	1.1×10^{-14}	2.7×10^{-6}	2.6×10^{-9}	3.7×10^{-6}

Definition of abbreviations: BMI = body mass index, COPD= chronic obstructive pulmonary disease, FEV = forced expiratory volume, FVC = forced vital capacity, N.A. = not applicable.

Statistical analysis was performed applying Mann Whitney test.

Table E2: List of confirmed significant metabolites from the non-targeted metabolomics platform for each comparison with the corresponding *p*-value, *q*-value, corrected *p*-value and fold change.

Metabolites	Smokers *vs.* COPD								
	♀♂			♀			♂		
	$p^{*}(q^{\dagger})$	$p^{\ddagger}$	FC	*p* (*q*)	*p*	FC	*p* (*q*)	*p*	FC
12(13)EpODE[§]	0.008 (0.06)	0.006	1.3	0.03 (0.2)	0.01	1.4	0.2 (0.5)	0.2	1.2
12-HETE[§]	<0.0001 (0.002)	0.0004	3.4	0.0005 (0.01)	0.004	4.7	0.02 (0.3)	0.06	2.5
15-HEDE[§]	0.01 (0.06)	0.03	1.3	0.2 (0.3)	0.2	1.3	0.02 (0.3)	0.07	1.4
17-β-estradiol-glucuronide[§]	0.01 (0.07)	0.01	1.9	0.04 (0.2)	0.2	2.2	0.2 (0.5)	0.03	1.7
4-HDoHE[§]	<0.0001 (0.0006)	<0.0001	2.9	0.0005 (0.01)	0.0001	3.2	0.002 (0.2)	0.006	2.7
5(6)-EpETrE[§]	0.01 (0.06)	0.3	1.3	0.3 (0.4)	0.4	1.1	0.007 (0.2)	0.03	1.5
5-Methoxyindoleacetate[§]	0.8 (0.5)	0.8	-1.1	0.06 (0.2)	0.05	2.7	0.03 (0.3)	0.01	-3.0
5'-Methylthioadenosine[§]	0.3 (0.3)	0.3	1.2	0.04 (0.2)	0.1	1.6	0.7 (0.6)	0.5	-1.0
AEA[§]	0.03 (0.1)	0.03	1.3	0.02 (0.1)	0.006	1.4	0.6 (0.5)	0.9	1.1
Allantoin[§]	0.02 (0.08)	0.07	1.3	0.02 (0.1)	0.1	1.3	0.3 (0.5)	0.3	1.3
Asp-Leu[**]	0.009 (0.06)	0.03	1.3	0.05 (0.2)	0.1	1.3	0.09 (0.4)	0.1	1.2
Carnitine(C10:0)[**]	0.06 (0.2)	0.02	1.3	0.1 (0.3)	0.008	1.4	0.4 (0.5)	0.7	1.3
Carnitine(C10:1)[**]	0.09 (0.2)	0.006	1.2	0.03 (0.2)	0.001	1.4	0.8 (0.6)	0.9	1.1
Carnitine(C12:0)[**]	<0.0001 (0.003)	0.0002	1.3	0.0004 (0.01)	<0.0001	1.4	0.07 (0.4)	0.2	1.1
Carnitine(C12:1)[**]	0.05 (0.1)	0.009	1.3	0.03 (0.2)	0.001	1.4	0.7 (0.6)	0.7	1.2
Carnitine(C14:0)[**]	0.0009 (0.01)	0.004	1.4	0.06 (0.2)	0.04	1.3	0.007 (0.2)	0.05	1.4
Carnitine(C14:0-OH)[**]	0.05 (0.1)	0.01	1.3	0.008 (0.07)	0.004	1.5	0.8 (0.6)	0.4	1.1
Carnitine(C14:1)[**]	0.005 (0.04)	0.004	1.7	0.05 (0.2)	0.002	2.4	0.05 (0.4)	0.2	1.3
Carnitine(C16:0)[§]	0.01 (0.06)	0.009	1.2	0.2 (0.3)	0.2	1.1	0.02 (0.3)	0.03	1.3
Carnitine(C16:1)[**]	0.003 (0.03)	0.0004	1.4	0.06 (0.2)	0.008	1.3	0.04 (0.4)	0.02	1.5
Carnitine(C18:0)[§]	0.02(0.1)	0.04	1.2	0.4 (0.4)	0.5	1.1	0.04 (0.3)	0.05	1.4
Carnitine(C18:0-OH)[**]	0.07 (0.2)	0.1	1.2	0.4 (0.4)	0.9	1.0	0.08 (0.8)	0.09	1.3
Carnitine(C18:1)[§]	0.0002 (0.005)	0.0001	1.6	0.03 (0.2)	0.02	1.3	0.002 (0.2)	0.002	1.8

Carnitine(C18:2)**	0.004 (0.04)	0.0008	1.4	0.04 (0.2)	0.007	1.3	0.05 (0.4)	0.02	1.5
Cortisone§	0.2 (0.3)	0.6	1.1	0.03 (0.1)	0.07	1.2	0.6 (0.5)	0.4	-1.0
Dityrosine**	0.0003 (0.006)	0.04	1.4	0.0002 (0.01)	0.08	1.6	0.1 (0.5)	0.3	1.2
Erythronicacid§	<0.0001 (0.001)	0.002	1.3	0.0002 (0.01)	0.0003	1.4	0.01 (0.3)	0.4	1.2
Estrone-glucuronide**	0.07 (0.2)	0.09	1.3	0.05 (0.2)	0.04	1.6	0.6 (0.5)	0.4	1.1
Glyco-chenodeoxycholate-S**	0.04 (0.1)	0.008	1.8	0.1 (0.3)	0.08	1.8	0.2 (0.5)	0.04	1.8
Gulose**	0.02 (0.1)	0.005	-1.3	0.2 (0.3)	0.1	-1.2	0.06 (0.4)	0.01	-1.4
Hippurate§	0.5 (0.4)	1.0	1.2	0.03 (0.2)	0.2	2.1	0.2 (0.5)	0.2	-1.5
Hypoxanthine§	0.002 (0.02)	0.03	1.2	0.004 (0.05)	0.03	1.8	0.1 (0.4)	0.3	-1.1
Inosine§	0.005 (0.04)	0.1	1.4	0.008 (0.07)	0.3	1.5	0.2 (0.5)	0.3	1.3
Isocitrate§	0.04 (0.1)	0.1	1.1	0.004 (0.05)	0.001	1.3	0.9 (0.6)	0.5	-1.0
Leu-Pro**	0.0008 (0.01)	0.002	-1.2	0.1 (0.3)	0.2	-1.1	0.001 (0.2)	0.002	-1.3
LysoPA(16:0) §	<0.0001 (0.001)	0.0004	2.0	<0.0001 (0.01)	0.0001	2.4	0.03 (0.3)	0.1	1.6
LysoPA(18:2)**	0.001 (0.02)	0.004	1.7	0.0003 (0.01)	0.002	2.1	0.2 (0.5)	0.1	1.4
LysoPC(15:0)**	0.003 (0.03)	0.01	1.3	0.001 (0.02)	0.001	1.4	0.2 (0.5)	0.4	1.2
LysoPC(16:0)§	0.02 (0.09)	0.005	1.3	0.08 (0.2)	0.04	1.2	0.1 (0.5)	0.05	1.4
LysoPC(18:2)**	0.8 (0.5)	0.01	1.2	0.6 (0.5)	0.008	1.4	0.8 (0.6)	0.7	1.0
LysoPE(16:1)**	0.01 (0.06)	0.03	1.2	0.05 (0.2)	0.03	1.3	0.1 (0.5)	0.3	1.2
Malate§	<0.0001 (0.002)	0.0003	1.7	0.0004 (0.01)	0.0002	2.0	0.008 (0.2)	0.06	1.5
Methylhippurate§	0.01 (0.06)	0.08	1.2	0.05 (0.2)	0.3	1.1	0.06 (0.4)	0.2	1.2
Myristoylglycine**	0.04 (0.1)	0.08	1.1	0.003 (0.04)	0.005	1.3	0.9 (0.6)	0.4	-1.1
N-methyl-D-aspartic acid§	0.01 (0.08)	0.05	1.3	0.1 (0.2)	0.5	1.2	0.09 (0.4)	0.05	1.3
N-Oleoyl-L-serine§	0.0003 (0.006)	0.0003	1.6	0.0002 (0.01)	0.0001	2.0	0.1 (0.5)	0.9	1.2
OEA§	0.04 (0.1)	0.03	1.2	0.02 (0.1)	0.005	1.3	0.5 (0.5)	0.7	1.1
OH-androstenone-S**	0.09 (0.2)	0.1	1.8	0.06 (0.2)	0.2	2.6	0.7 (0.6)	0.3	1.2
OH-cholestenone**	0.3 (0.3)	0.9	-1.2	0.02 (0.1)	0.03	-1.9	0.7 (0.6)	1	1.4
OH-hexadecanoic acid**	<0.0001 (0.003)	0.001	1.2	0.02 (0.1)	0.002	1.2	0.003 (0.2)	0.09	1.2

OH-octadecanoic acid**	0.005 (0.05)	0.0006	1.5	0.09 (0.2)	0.002	1.5	0.03 (0.3)	0.03	1.5
Oleic Acid§	0.01(0.07)	0.003	1.4	0.04 (0.2)	0.004	1.5	0.1 (0.5)	0.1	1.3
Palmitic Acid§	0.02 (0.09)	0.008	1.3	0.04 (0.2)	0.004	1.4	0.2 (0.5)	0.3	1.2
Palmitoleic Acid§	0.004 (0.04)	0.002	1.8	0.03 (0.2)	0.005	1.9	0.07 (0.4)	0.07	1.7
PC(14:1/15:0)**	0.03 (0.1)	0.07	1.7	0.03 (0.2)	0.02	1.8	0.3 (0.5)	0.8	1.7
PC(15:0/22:6)**	0.4 (0.4)	0.1	-1.1	0.2 (0.3)	0.04	-1.3	0.7 (0.6)	0.9	1.1
PC(16:1/P-18:1)**	0.002 (0.03)	0.2	-1.2	0.2 (0.3)	0.3	1.1	0.004 (0.2)	0.1	-1.7
PE(18:2/P-18:1)**	0.4 (0.4)	0.8	-1.1	0.8 (0.6)	0.3	1.2	0.03 (0.3)	0.1	-1.5
PEA§	0.02 (0.08)	0.2	1.4	0.04 (0.2)	0.002	1.4	0.2 (0.5)	0.2	1.4
Phenylpropionylglycine**	0.001 (0.02)	0.005	1.3	0.002 (0.04)	0.001	1.4	0.2 (0.5)	0.3	1.2
Sorbitol§	0.007 (0.06)	0.04	1.7	0.3 (0.4)	0.4	1.3	0.01 (0.2)	0.06	2.2
Sorbose§	0.03 (0.1)	0.02	-1.1	0.3 (0.4)	0.2	-1.0	0.04 (0.4)	0.04	-1.1
S-1P§	0.003 (0.03)	0.03	1.2	0.0004 (0.01)	0.02	1.3	0.3 (0.5)	0.3	1.1
Sn-1P§	0.01 (0.06)	0.2	1.1	0.0001 (0.01)	0.0009	1.3	1.0 (0.6)	0.8	1.0
Succinate§	<0.0001 (0.001)	0.0004	1.3	0.0003 (0.01)	0.0004	1.4	0.005 (0.2)	0.2	1.2
Succinyladenosine	0.04 (0.1)	0.2	1.1	0.05 (0.2)	0.2	1.2	0.3 (0.5)	0.6	1.1
Tryptophan§	0.04 (0.1)	0.03	-1.1	0.7 (0.5)	0.7	-1.0	0.01 (0.2)	0.005	-1.2
Uracil§	0.05 (0.1)	0.5	1.2	0.04 (0.2)	0.2	1.2	0.5 (0.5)	0.7	1.2

* = *p*-value from Mann Whitney test, † = Storey's *q* value, ‡ = *p*-value corrected for age & packyears, § = metabolites confirmed with standard & MS/MS, ** = metabolites confirmed with MS/MS

Definition of abbreviations: 12(13)EpODE = 12(13)-Epoxyoctadecadienoic acid, 12-HETE = 12-Hydroxyicosatetraenoic acid, 15-HEDE =15-Hydroxyeicosadienoic acid, 4-HDoHE = 4-Hydroxydocosahexaenoic acid, 5(6)-EpETrE = 5(6)-Epoxyeicosatrienoic acid, AEA = *N*-arachidonoylethanolamine, Asp-Leu = Aspartic acid-Leucine, FC = fold change, OH = Hydroxy, S = sulfate, Leu-Pro = Leucine-Proline, LysoPA = Lyso-phosphatidic acid. LysoPC = Lysophosphatidylcholine, LysoPE = Lysophosphatidylethanolamine, OEA = *N*-oleoylethanolamine, PC = Phosphatidylcholine, PE = Phosphatidylethanolamine, PEA = *N*-palmitoylethanolamide, S-1P = Sphingosine-1-phosphate, Sn-1P = Sphinganine-1-phosphate.

Table E3: Metabolite list from targeted Biocrates metabolomics analysis with concentration (µM) for each group and corresponding *p*-value and *q*-value.

Metabolites	Female Smoker	Female COPD	Male Smoker	Male COPD	Smoker *vs.* COPD		
					♀♂ *p**(*q*†)	♀ *p*(*q*)	♂ *p*(*q*)
Acetyl-ornithine	6.7±1.1	6.5±1.3	6.2±0.9	6.9±0.9	N.S.	N.S.	0.03(0.3)
Asymmetric dimethylarginine	0.5±0.1	0.5±0.1	0.4±0.1	0.5±0.1	N.S.	0.04(0.2)	N.S.
Alanine	349.9±77.5	393.8±95.9	347.4±77.9	385.7±70.8	0.05(0.1)	N.S.	N.S.
α-Aminoadipic acid	0.9±0.3	0.9±0.2	1.0±0.3	1.0±0.3	N.S.	N.S.	N.S.
Arginine	144.8±22.9	143.2±23.2	138.9±22.4	153.4±18.2	N.S.	N.S.	0.05(0.3)
Aspartate	21.7±4.9	26.6±5.3	22.3±4.8	26.9±3.8	0.001(0.02)	0.03(0.2)	0.01(0.3)
Carnitine (C0)	50.4±11.8	51.1±11.2	55.8±12.7	58.7±11.3	N.S.	N.S.	N.S.
Carnitine (C10:0)	0.3±0.1	0.4±0.2	0.4±0.1	0.5±0.2	0.009(0.05)	0.02(0.1)	N.S.
Carnitine (C10:1)	0.2±0.05	0.2±0.1	0.2±0.1	0.2±0.1	0.002(0.03)	0.002(0.05)	N.S.
Carnitine (C10:2)	0.05±0.01	0.1±0.01	0.1±0.01	0.1±0.02	0.01(0.06)	0.02(0.1)	N.S.
Carnitine (C12:0)	0.1±0.04	0.1±0.1	0.1±0.1	0.2±0.1	0.01(0.05)	0.01(0.1)	N.S.
Carnitine (C12:1)	0.1±0.04	0.2±0.1	0.2±0.1	0.2±0.1	0.001(0.02)	0.001(0.04)	N.S.
Carnitine (C12-DC)	0.1±0.01	0.1±0.005	0.1±0.01	0.1±0.01	N.S.	N.S.	N.S.
Carnitine (C14:0)	0.02±0.01	0.04±0.01	0.03±0.01	0.03±0.01	0.001(0.02)	0.001(0.03)	N.S.
Carnitine (C14:1)	0.05±0.01	0.1±0.02	0.1±0.02	0.1±0.02	0.001(0.02)	0.001(0.03)	N.S.
Carnitine (C14:1-OH)	0.01±0.002	0.01±0.003	0.01±0	0.01±0.01	N.S.	0.03(0.2)	N.S.
Carnitine (C14:2)	0.02±0.01	0.03±0.01	0.02±0.01	0.03±0.01	0.04(0.1)	0.02(0.1)	N.S.
Carnitine (C14:2-OH)	0.01±0.003	0.01±0.002	0.01±0	0.01±0.002	0.008(0.05)	0.04(0.2)	N.S.
Carnitine (C16)	0.1±0.02	0.1±0.02	0.1±0.05	0.1±0.03	0.003(0.03)	0.01(0.1)	N.S.
Carnitine (C16:1)	0.02±0.005	0.03±0.01	0.03±0.01	0.03±0.01	0.002(0.03)	0.01(0.1)	N.S.
Carnitine (C16:1-OH)	0.02±0.002	0.01±0.003	0.02±0	0.02±0.004	N.S.	N.S.	N.S.
Carnitine (C16:2)	0.02±0.01	0.02±0.01	0.02±0.01	0.02±0.01	0.008(0.05)	0.02(0.1)	N.S.

Carnitine (C16:2-OH)	0.02±0.004	0.01±0.004	0.02±0	0.02±0.003	0.004(0.04)	0.02(0.1)	N.S.
Carnitine (C16-OH)	0.02±0.01	0.02±0.01	0.02±0.01	0.02±0.01	0.001(0.02)	0.006(0.1)	N.S.
Carnitine (C18:0)	0.05±0.02	0.1±0.01	0.1±0.02	0.1±0.02	N.S.	N.S.	N.S.
Carnitine (C18:1)	0.1±0.03	0.1±0.03	0.1±0.05	0.1±0.04	0.03(0.1)	N.S.	N.S.
Carnitine (C18:1-OH)	0.01±0.002	0.01±0.003	0.01±0	0.01±0.002	0.01(0.06)	0.01(0.1)	N.S.
Carnitine (C18:2)	0.03±0.01	0.03±0.01	0.03±0.01	0.04±0.01	N.S.	N.S.	N.S.
Carnitine (C2:0)	5.4±2	6.1±2	6.2±2.5	7.5±4.2	N.S.	N.S.	N.S.
Carnitine (C3:0)	0.3±0.1	0.3±0.1	0.3±0.1	0.4±0.1	N.S.	N.S.	N.S.
Carnitine (C3:1)	0.01±0.001	0.01±0.001	0.01±0	0.02±0.002	N.S.	N.S.	N.S.
Carnitine (C3-DC/C4-OH)	0.04±0.02	0.1±0.03	0.1±0.04	0.1±0.1	0.03(0.1)	N.S.	N.S.
Carnitine (C3:0-OH)	0.02±0.004	0.02±0.004	0.02±0	0.02±0.004	N.S.	N.S.	N.S.
Carnitine (C4:0)	0.2±0.1	0.2±0.1	0.2±0.1	0.2±0.05	N.S.	N.S.	N.S.
Carnitine (C4:1)	0.03±0.01	0.02±0.004	0.03±0	0.02±0.003	0.006(0.04)	N.S.	0.02(0.3)
Carnitine (C5:0)	0.1±0.03	0.1±0.02	0.1±0.03	0.1±0.03	N.S.	N.S.	N.S.
Carnitine (C5:1)	0.1±0.01	0.05±0.01	0.1±0.01	0.05±0.02	0.01(0.06)	N.S.	N.S.
Carnitine (C5:1-DC)	0.04±0.01	0.03±0.01	0.04±0.01	0.04±0.01	0.005(0.04)	N.S.	0.03(0.3)
Carnitine (C5-DC/C6-OH)	0.03±0.01	0.03±0.004	0.03±0	0.03±0.004	N.S.	N.S.	N.S.
Carnitine (C5:0-M-DC)	0.04±0.02	0.04±0.01	0.04±0.01	0.04±0.01	N.S.	N.S.	N.S.
Carnitine (C5-OH/C3-DC-M)	0.03±0.01	0.03±0.01	0.03±0	0.03±0.004	N.S.	N.S.	N.S.
Carnitine (C6:0/C4:1-DC)	0.1±0.03	0.1±0.03	0.1±0.03	0.1±0.03	0.008(0.05)	0.03(0.1)	N.S.
Carnitine (C6:1)	0.03±0.01	0.02±0.01	0.03±0.01	0.03±0.01	0.006(0.04)	0.02(0.1)	N.S.
Carnitine (C7:0-DC)	0.03±0.01	0.04±0.02	0.04±0.01	0.04±0.02	N.S.	N.S.	N.S.
Carnitine (C8:0)	0.2±0.1	0.3±0.1	0.3±0.1	0.3±0.1	0.01(0.06)	0.03(0.1)	N.S.
Carnitine (C9:0)	0.03±0.01	0.03±0.01	0.03±0.01	0.04±0.02	0.05(0.1)	0.01(0.1)	N.S.
Citruline	38.8±10	41.4±6.7	39.4±9.7	36.6±9.1	N.S.	N.S.	N.S.
Creatinine	83.3±13.3	80.3±18.4	99.9±16	96.4±19.8	N.S.	N.S.	N.S.
Glutamine	645.7±69.2	718.7±108.6	681.6±116.4	702.1±128.8	N.S.	N.S.	N.S.
Glutamic acid	100.4±32.9	89.9±32.5	96.4±29.5	99.6±50.4	N.S.	N.S.	N.S.
Glycine	338.4±116.8	373.5±139.9	269.6±49.2	276.7±58.7	N.S.	N.S.	N.S.

Sugars	6086±818.1	6312±1225.1	6442.1±951.5	6230.1±1072.8	N.S.	N.S.	N.S.
Histidine	94.1±10.1	95.2±10.5	93.7±9.9	96.6±13.8	N.S.	N.S.	N.S.
Isoleucine	71.7±14.9	72.1±14.7	83.9±17.9	89.7±28.4	N.S.	N.S.	N.S.
Kynurenine	2.3±0.9	2.7±0.7	2.5±1	2.2±0.6	N.S.	N.S.	N.S.
Leucine	127.4±21.4	139.4±22.6	158.4±26.5	160.5±40.3	N.S.	0.04(0.2)	N.S.
Lysine	183.3±35.6	193.4±27.6	187.1±32.3	182.9±20.4	N.S.	N.S.	N.S.
LysoPC (C14:0)	5.8±1.1	6.2±0.7	5.5±0.8	5.8±0.8	0.04(0.1)	0.05(0.2)	N.S.
LysoPC (C16:0)	137.6±25.7	144.4±27.8	138.1±29.9	149.4±29.2	N.S.	N.S.	N.S.
LysoPC (C16:1)	4.7±2.3	5.1±1.5	4.3±1.8	5.9±3.1	0.05(0.1)	N.S.	N.S.
LysoPC (C17:0)	2.8±0.5	2.7±0.6	2.4±0.5	2.6±0.7	N.S.	N.S.	N.S.
LysoPC (C18:0)	42.2±7.6	44.6±7.9	41.5±8.6	45.1±8.7	N.S.	N.S.	N.S.
LysoPC (C18:1)	35.6±11.6	37.2±10.2	37.3±10.7	42.6±16.4	N.S.	N.S.	N.S.
LysoPC (C18:2)	43.5±23.8	39.4±10.6	48±15.3	43.2±11.1	N.S.	N.S.	N.S.
LysoPC (C20:3)	3.3±1	3.5±1	3.7±1.2	3.9±1.1	N.S.	N.S.	N.S.
LysoPC (C20:4)	7.2±1.5	7.6±2.2	8.8±2.8	9.3±2.9	N.S.	N.S.	N.S.
LysoPC (C24:0)	0.4±0.1	0.5±0.2	0.5±0.1	0.5±0.2	N.S.	N.S.	N.S.
LysoPC (C26:0)	0.6±0.3	0.7±0.3	0.7±0.3	0.8±0.6	N.S.	N.S.	N.S.
LysoPC (C26:1)	0.4±0.2	0.5±0.2	0.4±0.2	0.5±0.3	N.S.	N.S.	N.S.
LysoPC (C28:0)	0.7±0.2	0.8±0.3	0.7±0.2	0.7±0.4	N.S.	N.S.	N.S.
LysoPC (C28:1)	0.9±0.3	1±0.2	0.8±0.3	0.8±0.3	N.S.	N.S.	N.S.
Methiotine	24.2±3	25.9±5.4	29.2±4.8	28.4±7.7	N.S.	N.S.	N.S.
Methionine-Sulfoxide	1.2±0.7	0.8±0.7	1.1±0.6	0.7±0.3	0.006(0.04)	N.S.	N.S.
Ornithine	80.4±18.2	97.4±20.8	88.2±24	81.3±22.2	N.S.	0.008(0.1)	N.S.
PC (C24:0)	0.2±0.1	0.2±0.1	0.2±0.1	0.2±0.2	N.S.	N.S.	N.S.
PC (C26:0)	1.1±0.5	1.2±0.4	1.1±0.5	1.3±0.9	N.S.	N.S.	N.S.
PC (C28:1)	4.5±0.9	4.3±0.8	3.3±0.6	3.5±0.6	N.S.	N.S.	N.S.
PC (C30:0)	5.7±1.9	6.3±1.3	4.5±1.5	5±1.8	N.S.	N.S.	N.S.
PC (C32:0)	15.5±3.2	16.7±2.5	13.7±3.2	15.2±4.2	N.S.	N.S.	N.S.
PC (C32:1)	25.1±17.1	27.5±10.9	20.2±11.1	31.6±22.5	N.S.	N.S.	N.S.

PC (C32:3)	0.6±0.1	0.6±0.1	0.5±0.1	0.5±0.1	N.S.	N.S.	N.S.
PC (C34:1)	212.2±19	209±14.4	197.8±23.1	210.5±28.1	N.S.	N.S.	N.S.
PC (C34:2)	213.1±15.4	205.7±14.2	199.5±19.1	208.4±23.4	N.S.	N.S.	N.S.
PC (C34:3)	20.7±7.9	21.3±5.6	17±4	21.9±10.3	N.S.	N.S.	N.S.
PC (C34:4)	1.9±0.8	2.1±0.5	1.6±0.6	1.9±0.8	N.S.	N.S.	N.S.
PC (C36:0)	5.9±1.6	5.5±1.7	4.9±1.3	4.9±1.5	N.S.	N.S.	N.S.
PC (C36:1)	81.7±20	85.2±19.7	68.4±16.2	80.3±25.8	N.S.	N.S.	N.S.
PC (C36:2)	186.9±15.2	178.4±15.2	174.7±14.4	183.1±19.8	N.S.	N.S.	N.S.
PC (C36:3)	137.7±27.4	137.4±25.7	124.6±22.3	139.6±30.5	N.S.	N.S.	N.S.
PC (C36:4)	179.5±26.7	181.5±20.6	167.8±37.5	183±41.1	N.S.	N.S.	N.S.
PC (C36:5)	39.9±15.6	45.7±44.7	30±17.4	31.8±17	N.S.	N.S.	N.S.
PC (C36:6)	1.4±0.5	1.4±0.8	1±0.5	1.1±0.4	N.S.	N.S.	N.S.
PC (C38:0)	3.4±0.8	3.5±0.9	2.9±0.6	2.9±0.6	N.S.	N.S.	N.S.
PC (C38:3)	50.4±11.1	55.1±7.9	45.4±12.5	51.5±9.9	N.S.	N.S.	N.S.
PC (C38:4)	82.9±19.4	88.4±12.7	78.2±21.7	92±27.7	N.S.	N.S.	N.S.
PC (C38:5)	60.2±17.1	64.6±30.1	49.2±14.4	56.1±20.7	N.S.	N.S.	N.S.
PC (C38:6)	90.7±22.9	84.9±28.3	61.6±17.1	80.7±25	N.S.	N.S.	N.S.
PC (C40:1)	0.6±0.1	0.6±0.2	0.6±0.1	0.5±0.1	N.S.	N.S.	N.S.
PC (C40:2)	0.7±0.2	0.8±0.5	0.7±0.3	0.6±0.2	N.S.	N.S.	N.S.
PC (C40:3)	1±0.2	1.2±0.5	1±0.3	0.9±0.2	N.S.	N.S.	N.S.
PC (C40:4)	3.4±0.8	3.6±0.9	3.4±1	3.9±1.2	N.S.	N.S.	N.S.
PC (C40:5)	10.2±3	11±3.1	8.8±2.4	11±4.1	N.S.	N.S.	N.S.
PC (C40:6)	28.5±7.5	29.5±11.4	20.2±6.6	28.4±9.6	N.S.	N.S.	N.S.
PC (C42:0)	0.6±0.1	0.6±0.1	0.5±0.1	0.5±0.1	N.S.	N.S.	N.S.
PC (C42:1)	0.3±0.1	0.4±0.1	0.3±0.1	0.3±0.1	N.S.	N.S.	N.S.
PC (C42:2)	0.4±0.1	0.4±0.2	0.4±0.1	0.3±0.1	N.S.	N.S.	N.S.
PC (C42:4)	0.4±0.1	0.4±0.3	0.4±0.1	0.3±0.1	N.S.	N.S.	N.S.
PC (C42:5)	0.5±0.1	0.5±0.2	0.4±0.1	0.5±0.1	N.S.	N.S.	N.S.
PC (C42:6)	0.6±0.1	0.7±0.3	0.6±0.1	0.6±0.2	N.S.	N.S.	N.S.

PC (C30:0)	0.6±0.1	0.6±0.1	0.5±0.1	0.5±0.1	N.S.	N.S.	N.S.
PC (C30:1)	0.2±0.1	0.3±0.1	0.2±0.1	0.2±0.1	N.S.	N.S.	N.S.
PC (C30:2)	0.2±0.05	0.2±0.1	0.2±0.05	0.2±0.1	N.S.	N.S.	N.S.
PC (C32:1)	2.8±0.4	3±0.4	2.5±0.5	2.5±0.4	N.S.	N.S.	N.S.
PC (C32:2)	0.8±0.1	0.9±0.2	0.7±0.1	0.7±0.2	N.S.	N.S.	N.S.
PC (C34:0)	2.1±0.4	2.1±0.4	1.6±0.5	1.8±0.5	N.S.	N.S.	N.S.
PC (C34:1)	11.1±1.8	11.4±1.7	9.2±1.7	10.1±2.1	N.S.	N.S.	N.S.
PC (C34:2)	11.4±1.8	11.2±2.2	10.2±2.1	9.4±1.7	N.S.	N.S.	N.S.
PC (C34:3)	7.8±1.6	7.5±1.8	6.9±1.4	6.2±1.4	N.S.	N.S.	N.S.
PC (C36:0)	1.5±0.5	1.8±0.5	1.3±0.4	1.6±0.7	N.S.	N.S.	N.S.
PC (C36:1)	18.1±3.9	17.1±4.3	14.7±3.3	14.4±3.2	N.S.	N.S.	N.S.
PC (C36:2)	14.7±2.6	13.5±3	11.3±1.1	11.5±2	N.S.	N.S.	N.S.
PC (C36:3)	7.1±1.4	7.1±1.1	6.7±1.4	6.3±1.2	N.S.	N.S.	N.S.
PC (C36:4)	13.4±4.2	13.6±2.2	14±3.7	13.6±2.6	N.S.	N.S.	N.S.
PC (C36:5)	11.2±3	11.6±3.8	11±2.9	10.4±2	N.S.	N.S.	N.S.
PC (C38:0)	2.5±0.6	2.4±0.7	1.9±0.6	1.9±0.6	N.S.	N.S.	N.S.
PC (C38:1)	2±0.8	2.1±2.1	2.1±1.1	1.4±0.8	0.04(0.1)	N.S.	N.S.
PC (C38:2)	2.7±0.9	2.6±1.8	2.8±1	2.1±0.9	0.006(0.04)	N.S.	0.04(0.3)
PC (C38:3)	8.2±2.9	7.7±5	8±3.3	5.9±1.6	0.04(0.1)	N.S.	N.S.
PC (C38:4)	10.6±2.1	10.9±1.8	10.8±2.7	10±1.7	N.S.	N.S.	N.S.
PC (C38:5)	13.4±2.5	14.1±2.4	13.8±3.1	13.6±1.9	N.S.	N.S.	N.S.
PC (C38:6)	7.4±1.8	7.6±2.7	6.3±1.6	6.3±1.1	N.S.	N.S.	N.S.
PC (C40:1)	1.4±0.3	1.3±0.3	1.2±0.3	1.2±0.3	N.S.	N.S.	N.S.
PC (C40:2)	3.2±0.6	3±0.8	2.6±0.7	2.6±0.6	N.S.	N.S.	N.S.
PC (C40:3)	3±1.4	2.8±2.5	3.1±1.5	2±0.8	N.S.	N.S.	N.S.
PC (C40:4)	2.9±1.1	2.8±1.5	3.1±1.2	2.3±0.6	N.S.	N.S.	N.S.
PC (C40:5)	4.9±2	4.5±2.4	4.7±1.8	3.6±1	N.S.	N.S.	N.S.
PC (C40:6)	4.5±1	4.4±0.9	3.6±0.8	3.7±0.7	N.S.	N.S.	N.S.
PC (C42:0)	0.6±0.1	0.6±0.2	0.5±0.1	0.6±0.1	N.S.	N.S.	N.S.

PC (C42:1)	0.4±0.1	0.5±0.2	0.5±0.2	0.4±0.1	N.S.	N.S.	N.S.
PC (C42:2)	0.7±0.1	0.7±0.2	0.6±0.1	0.6±0.1	N.S.	N.S.	N.S.
PC (C42:3)	1±0.2	1±0.4	0.9±0.2	0.8±0.2	N.S.	N.S.	N.S.
PC (C42:4)	1±0.2	1.1±0.4	1±0.2	0.9±0.2	N.S.	N.S.	0.04(0.3)
PC (C42:5)	2.3±0.5	2.3±0.8	2.3±0.6	2±0.4	N.S.	N.S.	N.S.
PC (C44:3)	0.2±0.1	0.2±0.1	0.2±0.1	0.2±0.05	N.S.	N.S.	N.S.
PC (C44:4)	0.4±0.1	0.5±0.1	0.4±0.1	0.4±0.1	N.S.	N.S.	N.S.
PC (C44:5)	1.4±0.3	1.6±0.4	1.5±0.3	1.5±0.3	N.S.	N.S.	N.S.
PC (C44:6)	1.1±0.2	1.2±0.2	1±0.2	1±0.2	N.S.	N.S.	N.S.
Phenylalanine	72.8±11	78.6±12.8	81.5±9.7	82.6±9.2	N.S.	N.S.	N.S.
Proline	188.2±28.9	215.8±38.3	225.9±42.3	248.2±74	N.S.	N.S.	N.S.
Putrescine	0.1±0.05	0.2±0.04	0.2±0.04	0.2±0.1	N.S.	N.S.	N.S.
Sarcosine	18.3±3.8	19.8±3.4	18±3.1	20.1±3.3	0.05(0.1)	N.S.	N.S.
Symmetric dimethylarginine	0.6±0.1	0.6±0.1	0.6±0.1	0.6±0.1	N.S.	N.S.	N.S.
Serine	142.1±23.6	146±27.8	140.7±23.7	138.2±16.3	N.S.	N.S.	N.S.
Serotonin	0.8±0.4	0.9±0.5	0.8±0.2	0.9±0.3	N.S.	N.S.	N.S.
SM (C14:1-OH)	10.7±1.9	10.2±2.2	7.6±1.1	7.8±2.1	N.S.	N.S.	N.S.
SM (C16:1-OH)	6.9±1.4	6.8±1.4	5.1±0.7	5.1±1.3	N.S.	N.S.	N.S.
SM (C22:1-OH)	48.7±8	49.1±7.6	37.6±6.2	36.7±8.9	N.S.	N.S.	N.S.
SM (C22:2-OH)	58±9.5	58.4±11.1	41±5.8	41.9±8.6	N.S.	N.S.	N.S.
SM (C24:1-OH)	2.3±0.4	2.5±0.5	2±0.4	2±0.5	N.S.	N.S.	N.S.
SM (C16:0)	198.8±22.4	222.5±32.7	179.6±26.5	183.5±31.9	N.S.	N.S.	N.S.
SM (C16:1)	28.6±4.2	32±4.6	24.2±4	25±3.8	N.S.	0.05(0.2)	N.S.
SM (C18:0)	58.9±12	60.6±6.1	46.9±7.1	45.9±6.6	N.S.	N.S.	N.S.
SM (C18:1)	23.9±6	25.5±4.5	18.7±3.4	18.1±2.2	N.S.	N.S.	N.S.
SM (C20:2)	0.9±0.2	0.8±0.2	0.6±0.1	0.7±0.1	N.S.	N.S.	0.05(0.3)
SM (C24:0)	47.7±7.4	53.4±9.3	45.5±8.2	42.8±8.9	N.S.	N.S.	N.S.
SM (C24:1)	202.2±32.7	225.2±40.2	183.3±31.7	200.5±44.4	0.05(0.1)	N.S.	N.S.
SM (C26:0)	0.4±0.1	0.5±0.1	0.4±0.1	0.3±0.1	N.S.	N.S.	N.S.

SM (C26:1)	0.8±0.2	0.9±0.2	0.7±0.1	0.7±0.2	N.S.	N.S.	N.S.
trans4-OH-Proline	14.8±7.1	12.6±6.2	13.8±6.1	19.4±12.3	N.S.	N.S.	N.S.
Taurine	131.3±24.6	150.9±34.7	118.8±25.4	139.6±37.1	N.S.	N.S.	N.S.
Threonine	122±17.8	134.4±42.6	131.3±28.2	149.5±54.4	N.S.	N.S.	N.S.
Trptophan	90.7±20.6	82±13.4	94.9±14.1	78.9±10.2	0.003(0.03)	N.S.	0.001(0.04)
Tyrosine	73.3±12.4	81.3±14.6	83.4±14.9	81.3±11.8	N.S.	N.S.	N.S.
Valine	271.2±32.9	255.1±28.3	281.3±27.9	271.1±30.4	N.S.	N.S.	N.S.

* = *p*-value from Mann Whitney test, † = Storey's *q*-value, concentrations are presented as mean ± standard deviation
Definition of abbreviations: LysoPC= lysophosphatidylcholine, N.S. = not significant, OH= hydroxyl, PC= phosphatidylcholine, SM= sphingomyelin.

Data were acquired using the Biocrates AbsoluteIDQ p180 kit.

References:

1. Kohler M, Sandberg A, Kjellqvist S, Thomas A, Karimi R, Nyren S, Eklund A, Thevis M, Skold CM, Wheelock AM. Gender differences in the bronchoalveolar lavage cell proteome of patients with chronic obstructive pulmonary disease. *J Allergy Clin Immunol* 2013: 131(3): 743-751.

2. Forsslund H, Mikko M, Karimi R, Grunewald J, Wheelock AM, Wahlstrom J, Skold CM. Distribution of T-cell subsets in BAL fluid of patients with mild to moderate COPD depends on current smoking status and not airway obstruction. *Chest* 2014: 145(4): 711-722.

3. Karimi R, Tornling G, Forsslund H, Mikko M, Wheelock A, Nyren S, Skold CM. Lung density on high resolution computer tomography (HRCT) reflects degree of inflammation in smokers. *Respir Res* 2014: 15: 23.

4. Balgoma D, Yang M, Sjodin M, Snowden S, Karimi R, Levanen B, Merikallio H, Kaarteenaho R, Palmberg L, Larsson K, Erle DJ, Dahlen SE, Dahlen B, Skold CM, Wheelock AM, Wheelock CE. Linoleic acid-derived lipid mediators increase in a female-dominated subphenotype of COPD. *Eur Respir J* 2016: 47(6): 1645-1656.

5. Sandberg A, Skold CM, Grunewald J, Eklund A, Wheelock AM. Assessing recent smoking status by measuring exhaled carbon monoxide levels. *PLoS One* 2011: 6(12): e28864.

6. Eklund A, Blaschke E. Relationship between changed alveolar-capillary permeability and angiotensin converting enzyme activity in serum in sarcoidosis. *Thorax* 1986: 41(8): 629-634.

7. Löfdahl JM, Cederlund K, Nathell L, Eklund A, Sköld CM. Bronchoalveolar lavage in COPD: fluid recovery correlates with the degree of emphysema. *Eur Respir J* 2005: 25(2): 275-281.

8. Karimi R, Tornling G, Grunewald J, Eklund A, Skold CM. Cell recovery in bronchoalveolar lavage fluid in smokers is dependent on cumulative smoking history. *PloS one* 2012: 7(3): e34232.

9. Kamleh MA, Snowden SG, Grapov D, Blackburn GJ, Watson DG, Xu N, Stahle M, Wheelock CE. LC-MS Metabolomics of Psoriasis Patients Reveals Disease Severity-Dependent Increases in Circulating Amino Acids That Are Ameliorated by Anti-TNFalpha Treatment. *Journal of proteome research* 2015: 14(1): 557-566.

10. Wishart DS, Jewison T, Guo AC, Wilson M, Knox C, Liu Y, Djoumbou Y, Mandal R, Aziat F, Dong E, Bouatra S, Sinelnikov I, Arndt D, Xia J, Liu P, Yallou F, Bjorndahl T, Perez-Pineiro R, Eisner R, Allen F, Neveu V, Greiner R, Scalbert A. HMDB 3.0--The Human Metabolome Database in 2013. *Nucleic Acids Res* 2013: 41(Database issue): D801-807.

11. Gromski PS, Xu Y, Kotze HL, Correa E, Ellis DI, Armitage EG, Turner ML, Goodacre R. Influence of missing values substitutes on multivariate analysis of metabolomics data. *Metabolites* 2014: 4(2): 433-452.

12. Kirwan JA, Broadhurst DI, Davidson RL, Viant MR. Characterising and correcting batch variation in an automated direct infusion mass spectrometry (DIMS) metabolomics workflow. *Anal Bioanal Chem* 2013: 405(15): 5147-5157.

13. Wheelock AM, Wheelock CE. Trials and tribulations of 'omics data analysis: assessing quality of SIMCA-based multivariate models using examples from pulmonary medicine. *Mol Biosyst* 2013: 9(11): 2589-2596.

14. Eriksson L, Trygg J, Wold S. CV-ANOVA for significance testing of PLS and OPLS (R) models. *J Chemometr* 2008: 22(11-12): 594-600.

15. Levanen B, Bhakta NR, Torregrosa Paredes P, Barbeau R, Hiltbrunner S, Pollack JL, Skold CM, Svartengren M, Grunewald J, Gabrielsson S, Eklund A, Larsson BM, Woodruff PG, Erle DJ, Wheelock AM. Altered microRNA profiles in bronchoalveolar lavage fluid exosomes in asthmatic patients. *J Allergy Clin Immunol* 2013: 131(3): 894-903.

16. Trygg J, Gullberg J, Johansson AI, Jonsson P, Moritz T. Chemometrics in Metabolomics. *In:* Saito K, Dixon RA, Willmitzer L, eds. Plant Metabolomics (Biotechnology in Agriculture and Forestry 57). Springer Verlag, 2006.

17. Trygg J, Holmes E, Lundstedt T. Chemometrics in metabonomics. *J Proteome Res* 2007: 6(2): 469-479.

18. Eriksson L, Johansson E, Kettaneh-Wold N, Trygg J, Wikström C, Wold S. Multi- and Megavariate Data Analysis Part I Basic Principles and Applications. Umetrics AB, 2006.

19. Jackson JE. A Users Guide to Principal Components. John Wiley, New York, 1991.

20. Wold S, Ruhe A, Wold H, Dunn WJ. The collinearity problem in linear regression. The partial least squares approach to generalized inverses. *SIAM J Sci Stat Comput* 1984: 5(3): 735-743.

21. Wold S, Trygg J, Berglund A, Antti H. Some recent developments in PLS modeling. *Chemometr Intell Lab* 2001: 58(2): 131-150.

22. Trygg J, Wold S. Orthogonal projections to latent structures (O-PLS). *J Chemometr* 2002: 16(3): 119-128.

23. Bylesjo M, Rantalainen M, Cloarec O, Nicholson JK, Holmes E, Trygg J. OPLS discriminant analysis: combining the strengths of PLS-DA and SIMCA classification. *J Chemometr* 2006: 20(8-10): 341-351.

24. Wiklund S, Johansson E, Sjostrom L, Mellerowicz EJ, Edlund U, Shockcor JP, Gottfries J, Moritz T, Trygg J. Visualization of GC/TOF-MS-based metabolomics data for identification of biochemically interesting compounds using OPLS class models. *Analytical chemistry* 2008: 80(1): 115-122.

25. Trygg J, Wold S. Orthogonal Projections to Latent Structures (OPLS). *J Chemometr* 2002: 16(3): 119-128.

26. Wold S. Cross-Validatory Estimation of Number of Components in Factor and Principal Components Models. *Technometrics* 1978: 20(4): 397-405.

27. Cederkvist HR, Aastveit AH, Naes T. A comparison of methods for testing differences in predictive ability. *J Chemometr* 2005: 19(9): 500-509.

28. Eriksson L, Johansson E, Kettaneh-Wold N, Trygg J, Wikstrom C, Wold S. Multi- and megavariate data analysis. Umetrics AB, 2006.

29. Kohler M, Sandberg A, Kjellqvist S, Thomas A, Karimi R, Nyrén S, Thevis M, Eklund A, Skold CM, Wheelock AM. Gender differences in the bronchoalveolar lavage cell proteome of patients with COPD. *JACI* 2013: 131(3): 743-751.

30. Levänen B, Bhakta N, Paredes PT, Barbeau R, Pollack JL, Sköld CM, Svartengren M, Grunewald J, Gabrielsson S, Larsson BM, Eklund A, Woodruff P, Erle DJ, Wheelock ÅM. Differences in Exosomal MicroRNAs in Bronchoalveolar Lavage Fluid from Asthmatics and Healthy Individuals. *JACI* 2013: 131(3): 894-903.

31. Lundstrom SL, Levanen B, Nording M, Klepczynska-Nystrom A, Skold M, Haeggstrom JZ, Grunewald J, Svartengren M, Hammock BD, Larsson BM, Eklund A, Wheelock AM, Wheelock CE. Asthmatics exhibit altered oxylipin profiles compared to healthy individuals after subway air exposure. *PloS one* 2011: 6(8): e23864.

32. Lundstrom SL, Yang J, Kallberg HJ, Thunberg S, Gafvelin G, Haeggstrom JZ, Gronneberg R, Grunewald J, van Hage M, Hammock BD, Eklund A, Wheelock AM, Wheelock CE. Allergic asthmatics show divergent lipid mediator profiles from healthy controls both at baseline and following birch pollen provocation. *PloS one* 2012: 7(3): e33780.

33. Cohen J. What I have learned (so far). *American Psychologist* 1990: 45(12): 1304-1312.

34. Bylesjo M, Eriksson D, Sjodin A, Jansson S, Moritz T, Trygg J. Orthogonal projections to latent structures as a strategy for microarray data normalization. *BMC bioinformatics* 2007: 8: 207.

35. Blanc PD, Yen IH, Chen H, Katz PP, Earnest G, Balmes JR, Trupin L, Friedling N, Yelin EH, Eisner MD. Area-level socio-economic status and health status among adults with asthma and rhinitis. *The European respiratory journal : official journal of the European Society for Clinical Respiratory Physiology* 2006: 27(1): 85-94.

36. Saude EJ, Obiefuna IP, Somorjai RL, Ajamian F, Skappak C, Ahmad T, Dolenko BK, Sykes BD, Moqbel R, Adamko DJ. Metabolomic biomarkers in a model of asthma exacerbation: urine nuclear magnetic resonance. *American journal of respiratory and critical care medicine* 2009: 179(1): 25-34.

37. Saude EJ, Skappak CD, Regush S, Cook K, Ben-Zvi A, Becker A, Moqbel R, Sykes BD, Rowe BH, Adamko DJ. Metabolomic profiling of asthma: diagnostic utility of urine nuclear magnetic resonance spectroscopy. *The Journal of allergy and clinical immunology* 2011: 127(3): 757-764 e751-756.

38. Yorke J, Moosavi SH, Shuldham C, Jones PW. Quantification of dyspnoea using descriptors: development and initial testing of the Dyspnoea-12. *Thorax* 2010: 65(1): 21-26.

39. Smith J, Albert P, Bertella E, Lester J, Jack S, Calverley P. Qualitative aspects of breathlessness in health and disease. *Thorax* 2009: 64(8): 713-718.

40. Roy K, Smith J, Kolsum U, Borrill Z, Vestbo J, Singh D. COPD phenotype description using principal components analysis. *Respiratory research* 2009: 10: 41.

41. Burgel PR, Paillasseur JL, Caillaud D, Tillie-Leblond I, Chanez P, Escamilla R, Court-Fortune I, Perez T, Carre P, Roche N. Clinical COPD phenotypes: a novel approach using principal component and cluster analyses. *The European respiratory journal : official journal of the European Society for Clinical Respiratory Physiology* 2010: 36(3): 531-539.

42. Ubhi BK, Cheng KK, Dong J, Janowitz T, Jodrell D, Tal-Singer R, MacNee W, Lomas DA, Riley JH, Griffin JL, Connor SC. Targeted metabolomics identifies perturbations in amino acid metabolism that sub-classify patients with COPD. *Molecular bioSystems* 2012: 8(12): 3125-3133.

43. Ubhi BK, Riley JH, Shaw PA, Lomas DA, Tal-Singer R, MacNee W, Griffin JL, Connor SC. Metabolic profiling detects biomarkers of protein degradation in COPD patients. *Eur Respir J* 2012: 40(2): 345-355.

44. Forsslund H, Yang M, Mikko M, Karimi R, Nyren S, Engvall B, Grunewald J, Merikallio H, Kaarteenaho R, Wahlstrom J, Wheelock AM, Skold CM. Gender differences in the T-cell profiles of the airways in COPD associate with clinical phenotypes. *Int J Chron Obstruct Pulmon Dis* 2016: In press.

45. Duri S, Molthen RC, Tran CD. Discriminating pulmonary hypertension caused by monocrotaline toxicity from chronic hypoxia by near-infrared spectroscopy and multivariate methods of analysis. *Analytical biochemistry* 2009: 390(2): 155-164.

46. Silva E, Souchelnytskyi S, Kasuga K, Eklund A, Grunewald J, Wheelock AM. Quantitative intact proteomics investigations of alveolar macrophages in sarcoidosis. *The European respiratory journal : official journal of the European Society for Clinical Respiratory Physiology* 2012.

47. Lundström SL, Yang J, Källberg HJ, Thunberg S, Gafvelin G, Haeggström JZ, Grönneberg R, Grunewald J, van Hage M, Hammock BD, Eklund A, Wheelock ÅM, Wheelock CE. Allergic Asthmatics Show Divergent Lipid Mediator Profiles from Healthy Controls Both at Baseline and following Birch Pollen Provocation. *PloS one* 2012: 7(3): e33780.

48. Wheelock AM, Wheelock CE. Challenges in ‘omics investigations of respiratory disease: Assessing quality of SIMCA-based multivariate models. *Molecular bioSystems* 2013: submitted.
